# The Modulating Effects of Fruit and Fruit-Derived Interventions on Arterial Stiffness

**DOI:** 10.3390/nu18162627

**Published:** 2026-08-12

**Authors:** Marilyn S. Nehls

**Affiliations:** Department of Kinesiology and Health Promotion, University of Kentucky, Lexington, KY 40506, USA; mca243@uky.edu; Tel.: +1-(859)-257-2525

**Keywords:** vascular, polyphenols, antioxidative, inflammation, nitric oxide, anthocyanins, berries, hesperidin, L-citrulline

## Abstract

Fruit and fruit-derived interventions, when provided to various populations of interest, have the potential to contribute to cardioprotective benefits. Of particular interest, arterial stiffness serves as an important prognostic marker for cardiovascular disease and events, so many intervention studies have focused on the ability of fruit and fruit-derived interventions to improve arterial stiffness, especially in the aorta. Therefore, the aim of the present narrative review was to examine the available evidence on the relationship between fruit-based interventions and arterial stiffness. Five electronic databases were searched using a structured search strategy through December 2025. Among the 68 identified clinical investigations, findings were heterogeneous, although some studies reported improvements in interventions derived from berries, grapes, citrus fruits, and watermelon. Current evidence does not support favorable effects following interventions derived from tart cherry, and many fruits lack sufficient evidence to determine whether improvements in arterial stiffness are possible. Given the wide variety of polyphenols and bioactive compounds available in fruit and fruit-based interventions, there is strong theoretical potential for these interventions to exert beneficial effects on the cardiovascular system. Nevertheless, further research is warranted to understand whether fruit-based interventions can produce demonstrable changes in arterial stiffness among varying populations, and when such changes are observed, to elucidate the mechanisms underlying these effects. Thus, additional randomized controlled trials, especially with sufficient durations and adequate sample sizes to detect structural changes in the vascular system, are needed.

## 1. Introduction

Cardiovascular disease (CVD) persists as the leading cause of death globally, with 79.6% of CVD burden attributable to modifiable risk factors such as dietary risks [1]. One major hallmark of CVD development is the weakening of the vascular system, which is often characterized by arterial stiffening, among other things [2]. Arterial stiffness, particularly of the aorta, is a strong predictor of cardiovascular (CV) events independent of other CVD risk factors, including blood pressure [3,4]. Furthermore, aortic stiffness is a robust predictor of future hypertension, CV mortality, and all-cause mortality [4,5,6]. Thus, strategies that target arterial stiffness, especially of the aorta, may be of great value in decreasing the global burden of CVD.

Of particular interest, dietary risks have been identified as the second-leading attributable risk factor for CVD burden [1]. Furthermore, dietary interventions have been recognized as the most effectual interventions for improving both longevity and a healthful lifespan when considering more than a century of aging research [7]. Consequently, given their strong predictive power in determining CVD risk and future CV events [8,9,10], there has been great interest in dietary strategies to address arterial stiffness.

Previously, several epidemiological studies have shown that fruit consumption is associated with lower CVD risk [11,12,13,14]. Moreover, a cross-sectional study of 1898 women from the United Kingdom found that higher intakes of anthocyanins and flavones, polyphenols found in plant foods, were significantly associated with lower arterial stiffness and central blood pressure, and berry intake was further associated with lower arterial stiffness [15]. Polyphenols are bioactive compounds that have antioxidative and anti-inflammatory properties, with the ability to theoretically exert substantial benefits on the cardiovascular system [16]. This epidemiological data, as well as the supposed potent nature of polyphenols, has given rise to the study of a substantial number of fruit-based dietary interventions, encompassing whole fruits, juices, powders, and extracts, for arterial stiffness in different populations. While previous reviews have considered the effects of polyphenols on arterial stiffness [17] or have reviewed interventions of interest for a target population [9], no comprehensive review has synthesized the clinical evidence from randomized controlled trials evaluating fruit-based interventions on arterial stiffness. Therefore, the aim of this narrative review is to examine the current evidence among randomized controlled clinical trials that evaluate the efficacy of whole fruits and fruit-based interventions to improve arterial stiffness.

## 2. Materials and Methods

This narrative review employed a structured literature search to identify randomized controlled trials evaluating the effects of fruit-based interventions on arterial stiffness. PubMed, Embase, Web of Science, CINAHL, and the Cochrane Library were searched through December 2025 using a search strategy developed around terms related to fruit (e.g., fruit, berries, grapes, citrus, watermelon, and polyphenols) and arterial stiffness outcomes (e.g., arterial stiffness, pulse wave velocity, augmentation index, and vascular stiffness). The complete search strategy and search terms are provided in the Supplementary Materials. All relevant conference abstracts were examined and included if the study findings were not subsequently published as peer-reviewed journal articles. When a conference abstract located through the search strategy led to an identified published peer-reviewed journal article, the published article was included instead. Eligibility for inclusion was determined through the identification of trials that: (1) were randomized controlled trials; (2) evaluated an intervention involving whole fruits or fruit-derived interventions, including dried fruit, fruit juices, powders, concentrates, or extracts; (3) measured at least one arterial stiffness outcome; (4) were conducted in humans; and (5) were published in English. Studies were excluded if they: (1) only reported the effects of the intervention on acute exercise or postprandial conditions; or (2) did not include at least one study arm that tested a fruit-based intervention alone. Identified studies were screened manually by title and abstract for relevance using Rayyan’s review management system [18], and the remaining studies underwent full-text review to determine whether they met the inclusion and exclusion criteria. Due to the narrative nature of this review, studies were evaluated qualitatively rather than quantitatively, with a final summary of the strength of the evidence according to the following categories: **Strong** evidence indicates multiple RCTs demonstrated consistent effects on validated arterial stiffness markers, especially the gold-standard measure, cfPWV. **Moderate** evidence indicates multiple RCTs or one larger RCT of higher quality demonstrated improvements in cfPWV, although findings may be inconsistent, or multiple studies show replicated improvements among alternate measures of arterial stiffness. **Limited** evidence indicates preliminary evidence of positive findings from smaller trials or isolated positive findings in alternate measures of arterial stiffness. **Insufficient** evidence indicates that available studies are too limited, heterogeneous, or inconsistent.

## 3. Arterial Stiffness as a Clinical Marker

Arterial stiffness occurs with aging and various disease states and is marked by a progressive decline in arterial compliance. The capacity of the arteries to expand and recoil throughout the full cardiac cycle is indicative of current CV health [19]. Structural changes in the arteries associated with the stiffening process include decreased elasticity [20] due to changes in the structural proteins (collagen and elastin), calcification of the arteries, advanced glycation end-products, inflammation, oxidative stress, and matrix metalloproteinase activity [21,22,23]. These modifications result in the pulse pressure wave traveling at a faster velocity in stiffer arteries.

The gold standard methodology for determining arterial stiffness is the carotid-femoral pulse wave velocity (cfPWV), a regional measure of aortic arterial stiffness [24,25]. For cfPWV, a calculation of the velocity of the pulse pressure wave (distance divided by time, reported as meters per second) is determined by capturing measurements of the pulse pressure wave at the carotid and femoral sites. The resultant cfPWV value serves as a strong, independent predictor of future CV events [4,26,27], where a 1.0 m/s increase is associated with a 14% increase in CV events and a 15% increase in CV mortality. Accordingly, due to the superiority of cfPWV as a clinical marker of arterial stiffness, it is given the greatest weight throughout this review.

Other regional pulse wave velocity (PWV) measures are available and may offer some insight into arterial stiffness, but they should not be interpreted as interchangeable with cfPWV. Brachial-ankle pulse wave velocity (baPWV) is positively correlated with cfPWV [28], but it may serve as a better standalone marker of CVD risk than as a surrogate marker of cfPWV due to its dependence on the muscular arteries of the lower extremities in addition to the aorta [16]. Femoral-ankle pulse wave velocity (faPWV) primarily reflects lower-extremity peripheral arterial stiffness and serves as an independent predictor of CVD risk [29], whereas carotid-radial pulse wave velocity (crPWV) assesses upper-extremity arterial stiffness but may not be an independent predictor of CVD risk [30]. Brachial-knee pulse wave velocity (bkPWV) has been utilized occasionally [31,32], but it has not yet been validated for widespread clinical use. The Arteriograph24^TM^ utilizes oscillometric occlusion techniques to obtain “aortic PWV” for 24 h, but the inability to obtain sufficient measurements throughout the monitoring period raises questions about its clinical applicability [33]. Therefore, while various PWV methods exist, higher confidence should be placed on studies that utilize the gold standard cfPWV measure.

Indirect surrogate measures derived by pulse wave analysis (PWA), such as central pulse pressure (cPP), central blood pressure (cBP), augmentation pressure (AP), and augmentation index (AIx), provide complementary information. However, they are classified as systemic measures and should not be used interchangeably with cfPWV, as they reflect different physiological phenomena [24]. Brachial pulse pressure (bPP) has been utilized as a surrogate marker of arterial compliance [34], but cPP derived through PWA is a superior predictor of CV events than bPP [35]. Likewise, cSBP and cDBP, also derived through PWA, are more strongly associated with CV events than bSBP and bDBP [36]. Moreover, AP and AIx, often corrected to a heart rate of 75 beats per minute (AIx@HR75) [37], independently predict CV events and are commonly reported alongside PWV values. While this practice is recommended by expert consensus, these measures should not be considered interchangeable with cfPWV or interpreted as direct measures of arterial stiffness [24].

Additional arterial stiffness measures, including cardio-ankle vascular index (CAVI), digital volume pulse indices, and local stiffness measures derived from bioelectrical impedance, have also been used in intervention studies. CAVI tends to be favored over cfPWV in Asian countries and successfully predicts CV events in high-risk patients [38], but it incorporates muscular arterial segments in addition to the aorta in its derivation [39]. The digital volume pulse-stiffness index (DVP-SI) measure closely correlates with cfPWV [40], but the digital volume pulse-reflective index (DVP-RI) only appears to be predictive of CV events in men, not women [41]. A final local arterial stiffness measure using bioelectrical impedance technology is significantly correlated with PWV, though it has not gained much traction [42]. These measures of arterial stiffness have all been utilized in clinical trials that consider fruit for its ability to affect the vascular system. However, because the different measures of arterial stiffness reflect distinct physiological properties, the greatest emphasis is placed on findings that reflect a change in cfPWV due to its designation as the gold standard methodology.

## 4. Fruits, Fruit Juices, Fruit Extracts, and Fruit-Based Interventions of Interest for Arterial Stiffness

A total of 68 clinical intervention studies assessing the effects of fruits and fruit-based products on arterial stiffness were identified and categorized into 7 groups: blueberries, other berries, tart cherries, grapes, citrus, watermelon, and other fruits.

### 4.1. Blueberries and Blueberry-Based Interventions

A total of 13 identified clinical studies evaluated the effects of blueberries on arterial stiffness (Table 1). These studies ranged in duration from a single acute dose to a 6-month intervention. Across the studies, freeze-dried blueberry powder was the most common form of administration, but additional forms of administration included frozen blueberries, fresh blueberries, blueberry mousse, and blueberry juice. Aortic stiffness, as determined by cfPWV, remained unchanged in all blueberry studies that measured this outcome. However, baPWV decreased following 8 weeks of freeze-dried blueberry powder supplementation in postmenopausal women with pre- or stage 1 hypertension with a significant group-by-time interaction [43]. Additional studies involving young men and women [44] or generally healthy normal- and overweight men and women aged 18–60 years [45] found no improvements in baPWV after a single acute dose or crPWV after 1 week of blueberry administration, respectively. In the longest randomized controlled trial (RCT) to date for blueberries, a group of 50–75-year-old overweight and obese adults with MetS experienced a significantly lower AIx@HR75 after 6 months of consuming 1 cup of blueberries daily compared to ½ cup daily consumption and the placebo intervention [46]. Reductions in AIx and cSBP in the treatment group were also observed in a group of postmenopausal women and 18–50-year-old men given 6 weeks of freeze-dried blueberry powder; however, no significant group-by-time interaction was detected [47]. Central PP was significantly improved in the treatment group of postmenopausal women with pre- and stage 1 hypertension given freeze-dried blueberry powder at 4 weeks of a 12-week intervention, but the improvements did not persist to the 8-week or 12-week marks, and no significant group-by-time interaction was observed [48]. Thus, in the 13 studies, blueberries were found to be efficacious in improving at least one aspect of arterial stiffness among the treatment group within 5 of the clinical trials, but the effects were only robust enough to produce group-by-time interaction in one of the trials, and the gold standard marker of aortic stiffness, cfPWV, remained unchanged.

### 4.2. Other Berries and Other Berry-Based Interventions

While blueberries have prevailed as the most studied berry for potential improvements in vascular health, aronia berry, black and red raspberries, blackberries, blackcurrant, cranberries, strawberries, and a mixed red berry intervention have also been investigated (Table 2), amounting to a total of 13 studies ranging in duration from a single acute dose to 12 weeks. Within these studies, double-strength cranberry juice and CurraNZ^®^ blackcurrant extract were the only interventions that showed a group-by-time interaction for cfPWV [56,57]. The double-strength cranberry juice resulted in a 0.5 m/s reduction in cfPWV after a 4-week intervention in men and women with stable coronary artery disease [57]. The CurraNZ^®^ blackcurrant extract resulted in a reduction in cfPWV and cSBP following a 7-day intervention in men and women ≥65 years old; additionally, AIx was significantly lower than baseline in the treatment group despite no group-by-time interaction [58]. Furthermore, other significant changes in PWV were found, though not in the gold standard cfPWV measurement. Prehypertensive men and women had lower awake PWV after 12 weeks of 500 mg Aronox^®^ aronia berry extract compared to the control, along with a reduction in 24 h brachial and aortic AIx and awake brachial and aortic AIx, but these reductions did not persist 24 h after the last dose [59]. Postmenopausal women with pre- and stage 1 hypertension experienced a reduction from baseline in baPWV and faPWV after 8 weeks of 25 g of freeze-dried strawberry daily, but not the higher dose of 50 g of freeze-dried strawberry daily [60]. A dried black raspberry powder for 12 weeks was effective at lowering AIx in the treatment group among men and women with metabolic syndrome such that the change in AIx for the treatment group was significantly different from the change for the control group [61]. Additionally, cranberry juice successfully decreased AIx as well as cSBP at differing time points, though not all time points, after a single acute dose of cranberry juice with 3 different amounts of polyphenol content [62]. Lastly, while only a main effect of time for PWV was found, a mixed red berry fruit juice decreased peripheral resistance in prediabetic men and women after a 2-week intervention compared to the placebo with a significant group-by-time interaction [63]. Therefore, while no more than 4 studies exist for any one berry outside of blueberries, early evidence suggests some potential for other berries to improve measures of arterial stiffness.

### 4.3. Tart Cherries and Tart Cherry-Based Interventions

Tart cherries, particularly of the Montmorency variety, have received attention due to many suspected health benefits (Table 3). Eight studies have considered their effects on arterial stiffness, with the duration of intervention ranging from a single acute dose to 3 months. Investigations in tart cherries have not produced significant changes in cfPWV or other PWV measurements at alternate sites, although one study noted a main effect for time in cfPWV, as well as DVP-RI, but not a group or group-by-time interaction [70]. While an acute main effect of condition was detected in AIx@HR75 after a single dose of tart cherry concentrate in men and women with metabolic syndrome, this effect was not found after 6 days of administering the supplement [71]. Another investigation detected a main effect for time in AIx, AIx@HR75, and AP after a single acute dose of Montmorency tart cherry concentrate in men and women with metabolic syndrome, though no group or group-by-time interactions were found; post hoc analyses detected a significant difference between the placebo and intervention at 2 h post-consumption in AIx and AP, but no difference was apparent at 30 min, 1 h, 3 h, 4 h, or 5 h post-consumption [72]. Likewise, a study exploring the effects of a single acute dose of Montmorency tart cherry concentrate on healthy men and women identified a main effect for time in cSBP and cDBP, but no treatment or group-by-time interactions were demonstrated [73]. Consequently, of the 8 studies, no group-by-time interactions were detected in any of the arterial stiffness outcomes measured in the tart cherry investigations.

### 4.4. Grape-Based Interventions

Six studies have considered the effects of grapes and grape-based interventions on arterial stiffness using anywhere from a single acute dose to 12 weeks of intervention (Table 4). Of these studies, one study considering Concord grape juice for 14 days in men and women smokers resulted in a treatment effect in gold standard cfPWV compared to baseline, but no group-by-time interaction was found; furthermore, the treatment prevented an acute rise in cfPWV associated with smoking [77]. Similarly, a 12-week grape seed proanthocyanidin extract intervention caused a reduction in PWV from baseline in prehypertensive men and women at the 8-week and 12-week visits compared to baseline, but no group-by-time interaction followed [78]. These studies did not consider the effects on AIx or cBP, and no treatment effect or group-by-time interactions were found in measures of bPP [79] or bioelectrical impedance analysis [80]. Thus, although AIx and cBP have not yet been evaluated, preliminary findings suggest grape-based interventions may improve PWV.

### 4.5. Citrus-Based Interventions

Citrus-based interventions, typically administered as some form of citrus juice, were evaluated in 5 studies ranging in duration from an acute dose to 6 months (Table 5). Following a 6-month grapefruit juice intervention, healthy postmenopausal women experienced lower cfPWV measures in the treatment group compared to the placebo group after adjusting for baseline values, in a group-by-time interaction [83]. Young to older adults consuming concentrated β-cryptoxanthin-rich Satsuma mandarin juice for 12 weeks exhibited no significant group-by-time interaction in baPWV [84]. However, the post-intervention baPWV increased in both the treatment and control groups at the 8-month follow-up visit, indicating stiffer arteries in both groups, yet there was a significant group-by-time interaction that resulted in a lower baPWV in the treatment group [84]. An additional study considering the effects of 12 weeks of orange juice or hesperidin-enriched orange juice among men and women with pre- or stage 1 hypertension found that bPP decreased as a linear trend in a dose-dependent manner at 2 weeks, 6 weeks, and 10 weeks, but not 12 weeks; moreover, an acute dose of the hesperidin-enriched orange juice produced a significant reduction in bPP at 2 h, 4 h, and 6 h post-consumption [85]. No significant differences were found in AIx@HR75 [83,86] or cBP [87] in the few studies that considered these outcomes. Hence, while the citrus-based interventions were heterogeneous among the 5 studies, citrus-based interventions have demonstrated significant effects on markers of arterial stiffness that warrant further investigation.

### 4.6. Watermelon-Based Interventions

Five studies have evaluated the impact of watermelon, either as freeze-dried watermelon extract or watermelon juice, on arterial stiffness, from durations as short as an acute dose to as long as 6 weeks (Table 6). While no changes have been reported in the gold standard marker of arterial stiffness, cfPWV, to date, a 6-week study examining the effects of freeze-dried watermelon extract in postmenopausal women demonstrated a group-by-time interaction that resulted in a lower baPWV, in addition to cSBP and cDBP [88]. Also, an acute dose of wild watermelon juice in young, healthy women produced a significant group-by-time interaction in faPWV, though the interaction was attributed primarily to a large reduction at 30 min that did not persist at a significantly lower level at 60 or 90 min post-intervention [89]. A significant group-by-time interaction resulted in lower AIx in the intervention group among middle-aged prehypertensive and stage 1 hypertensive men and women consuming freeze-dried watermelon extract for 5 weeks [90]. A final investigation reported a significantly larger difference between the treatment and control groups in both AIx and AIx@HR75, as well as bPP, cPP, and cSBP, at 6 weeks than at baseline [91]. Consequently, the majority of watermelon intervention studies have found a significant effect on at least one marker of arterial stiffness or central hemodynamics.

### 4.7. Other Fruits and Fruit-Based Interventions

As seen in Table 7, this review identified 18 additional studies related to fruit-based interventions for arterial stiffness, with only 1–4 studies published on the fruit of choice, including apples, avocados, dragon fruit, the fruit of Acanthopanax senticosus Harms, olives, pears, pomegranates, plums, tomatoes, and mixed fruit interventions. The duration of these interventions ranged from a single acute dose to one year. When considering the impact of these interventions on the gold standard arterial stiffness marker, cfPWV, freeze-dried dragon fruit produced a group-by-time interaction in healthy men and women 3 h post-consumption after a single acute dose, but this effect was not maintained after the 14-day intervention; surprisingly, AIx@HR75 showed the opposite pattern, with no acute changes but a 7% decrease in the treatment group relative to the placebo group after the 14-day intervention [93]. Additionally, Pomonox^®^ P30, a concentrated pomegranate extract, given to young and middle-aged men and women for 28 days caused a significant reduction in cfPWV in the treatment group compared to baseline, but no group-by-time interaction was apparent [94]. Although no further investigations found improvements in cfPWV, only 6 considered this outcome. One investigation demonstrated a group-by-time interaction in baPWV following 12 weeks of intervention with Acanthopanax senticosus Harms fruit extract in men and women with elevated CVD risk among those given the low dose (500 mg) but not the high dose (1000 mg) [95]. Additionally, another study considering the effects of 4 weeks of pomegranate juice administered to overweight and obese men and women reported a decrease in PWV measured by Vicorder at 2 weeks and 4 weeks compared to baseline, though no group-by-time interaction was evident [96]. An improvement in AIx, namely an 8% reduction relative to the placebo over a 24 h time course, was noted in a subgroup of 6 subjects administered a single acute dose of Fruitflow^®^ tomato powder and tested for this secondary outcome in a group of apparently healthy men [97]. Among the full treatment group in this same investigation (*n* = 12), Fruitflow^®^ tomato powder decreased bPP relative to the placebo (*n* = 12) [97]. Furthermore, fresh pear [98] and pomegranate juice [99] caused significant reductions in bPP in the treatment groups from baseline among men and women with metabolic syndrome given the 12-week intervention and among men and women on chronic dialysis treatment given the intervention for one year, respectively. None of the 18 studies on various fruits found changes in cBP. Taken together, although limited evidence exists for each of the fruits located in Table 7, some encouraging findings have emerged for a few of the interventions.

### 4.8. Summary

For each fruit category, Table 8 summarizes the primary bioactive components, whether beneficial effects have been found in different arterial stiffness measures, and the strength of the current evidence.

## 5. Discussion

This narrative review of RCTs reveals many fruit-based interventions published over the past 16 years that were designed to alter arterial stiffness, with inconsistent findings. Notably, though this review evaluated fruit-based interventions, many of the identified studies considered concentrated products, such as fruit juices, powders, and extracts, instead of whole fruit. Therefore, the findings cannot be directly extrapolated to dietary whole fruit consumption. Given the evidence for improving central arterial stiffness, as measured by cfPWV, with a group-by-time interaction following chronic supplementation, double-strength cranberry juice [57], blackcurrant extract [58], and grapefruit juice [83] may hold the most promise in improving arterial stiffness. Additionally, support for chronic supplementation with freeze-dried blueberry [43], freeze-dried watermelon [88], and Acanthopanax senticosus Harms fruit extract [95] exists based on their capacity to decrease baPWV, a marker of both central and peripheral arterial stiffness. Favorable changes in central hemodynamic indices AIx or AIx@HR75 have been reported following chronic interventions with black raspberry extract [61], freeze-dried watermelon [90,91], freeze-dried blueberry [46], and freeze-dried dragon fruit [93] and an acute intervention with tomato powder [97]. Furthermore, chronic supplementation with freeze-dried watermelon has shown potential to improve additional hemodynamic measures, cSBP [88,91], cDBP [88], cPP [91], and bPP [91]. Other reported hemodynamic effects from these fruit-based interventions included a reduction in cDBP from chronic blackcurrant extract supplementation [58] and an acute decrease in bPP from tomato powder supplementation [97]. Nevertheless, in most cases, these results have not been replicated in, or extended to, other populations, warranting further investigation. More specifically, other than the hemodynamic changes following freeze-dried watermelon supplementation [88,91], these results are based on a single study and are, therefore, preliminary in nature. As such, they should be viewed as hypothesis-generating rather than definitive evidence.

Blueberries have been the most studied intervention (Table 1), and they have yet to produce changes in the gold standard central arterial stiffness marker, cfPWV, although group-by-time interactions were found in baPWV after 8 weeks in postmenopausal women with pre- or stage 1 hypertension [43]. Because baPWV is influenced by the muscular arteries of the lower limbs [28], it is possible that the effects of blueberries may be more potent on the smaller muscular arteries than the large, central elastic arteries, such as the aorta. However, it is also probable that the duration of the intervention was long enough to exert effects on the smaller muscular arteries but not the large elastic arteries. It is worth noting that 6 out of the 13 studies evaluated only an acute, single-dose intervention. The current duration for dietary interventions to elicit a change in arterial stiffness is unknown, although more robust interventions that include caloric restriction leading to weight loss or a complete alteration of dietary patterns might produce a more profound change in less time [111,112]. It has been suggested that when exercise-based interventions are utilized, at least 3 months of intervention may be necessary to alter aortic stiffness [113]. Only one blueberry study surpassed this 3-month threshold with a 6-month intervention and found a benefit of freeze-dried blueberry powder on AIx@HR75 but not cfPWV in overweight and obese adults 50–75 years old with MetS [46], but 2 additional studies were close to meeting this duration by intervening for 12 weeks and did not find any changes in arterial stiffness markers [48,49]. However, this difference in findings could be attributable to either the study duration or the population of interest. Therefore, the current evidence for blueberries to alter arterial structure in relatively short durations is not strong within the studied populations, but longer-term durations might affect arterial stiffness, and baPWV and AIx@HR75 might be more responsive to change than cfPWV.

Though promising results have been found in other berry-based interventions, no more than 4 studies have been published on a single berry, making current results preliminary. Of the 13 published berry studies (Table 2), none have met or surpassed the aforementioned 3-month mark, although 3 studies were close with 12-week interventions [59,61,64]. Nevertheless, benefits were seen in the gold standard cfPWV marker in 2 of the berry-based interventions, double-strength cranberry juice after 4 weeks in men and women with stable coronary artery disease [57] and CurraNZ^®^ blackcurrant extract after 7 days in older men and women [58]. To date, these results have not been tested or confirmed in other populations, although additional cranberry-based formulations have been attempted, showing a lack of comparable reduction in cfPWV after an acute intervention [62], an intervention of a similar 1-month length [66], or a longer 8-week intervention [67]. The 4-week intervention may have been more efficacious than these other interventions due to the cranberry juice being double-strength or due to the specific populations being tested. Only one of the 12-week interventions, the dried black raspberry powder in men and women with metabolic syndrome, produced benefits in arterial stiffness, with the change in AIx being larger for the treatment group than the control group [61]. Thus, there is currently not enough evidence to support a robust ability of these berries to improve arterial stiffness, but potential exists based on the studies described herein for double-strength cranberry juice, CurraNZ® blackcurrant extract, and dried black raspberry powder.

Of all interventions evaluated in this review, tart cherries and grapes seem the least likely to produce meaningful changes in arterial stiffness, though the longest intervention described was 3 months. It is unknown whether 3 months is a sufficient length for nutritional interventions to produce changes in arterial stiffening, so the possibility that tart cherries or grapes could exert benefits if administered for a longer duration cannot be excluded. Nevertheless, tart cherries remain the second-most-studied intervention for arterial stiffness in this review (Table 3), with 8 total studies, and no study has found a single group-by-time interaction for any arterial stiffness marker. On the other hand, grapes are the third-most-studied intervention (Table 4), with 6 total studies, and while favorable effects have been found in the treatment group for cfPWV and PWV [77,78], there may be no true treatment effect due to the changes not exceeding those found in the placebo group. However, for grapes, it should be noted that studies have not considered AIx or cBP as primary endpoints, and the longest study has been 12 weeks long, so it is unknown whether grapes could be capable of improving these additional outcomes or if a longer duration would produce changes. Given these reports, tart cherries and grapes appear to be ineffective interventions for modulating arterial stiffness when administered for shorter durations.

Interventions focused on citrus have been primarily based around juices, with a couple positive findings (Table 5). Although only 5 studies have been conducted, the longest intervention to date with citrus produced a significant positive finding in cfPWV following a 6-month grapefruit juice intervention in postmenopausal women [83]. None of the other studies have surpassed the 3-month mark, but 2 came close with a 12-week duration [84,85]. Within these 2 studies, the concentrated β-cryptoxanthin-rich Satsuma mandarin juice intervention may have curbed baPWV from increasing over time as much as it would have without the intervention [84], and the orange juice intervention demonstrated a linear trend in bPP in a dose-dependent manner based on hesperidin content [85]. Conversely, the shorter interventions, either acutely or for 28 days, yielded no beneficial effects [86,87]. Therefore, it might be postulated that citrus-based interventions may need to be of sufficient duration to confer benefits in arterial stiffness, but this hypothesis would need to be confirmed with future studies. Some caution should be exercised in applying fruit juice-based interventions due to a previous observational study suggesting a positive association between fruit juice consumption and cBP, though major limitations of this work include its cross-sectional nature and the lack of distinction between different kinds of fruit juice [114].

Of the available interventions in this review, watermelon exhibits the most potential for cBP-lowering effects (Table 6). Significant findings have been reported for watermelon with respect to cSBP [88,91], cDBP [88], cPP [91], and bPP [91]. Additionally, 2 studies have led to improvements in AIx [90,91] while one resulted in improvements in baPWV [88]. However, of the 5 studies that have explored potential benefits of watermelon for arterial stiffness, no study is longer than 6 weeks, significantly less than the 3-month mark. Thus, it might not be surprising that cfPWV has remained unaffected by watermelon interventions, though only 2 studies have considered this endpoint [89,91], and one of those studies utilized merely a single acute dose [89]. Overall, watermelon interventions have produced promising effects for cBP and arterial wave reflections after relatively short durations, but more studies need to consider longer-term interventions with watermelon to determine whether other aspects of arterial stiffness might be affected.

Beyond the above interventions, the 18 studies on a variety of other fruit-based interventions have revealed only a few findings that produced an interaction (Table 7). While the longest trial (one year) can be found among this group of interventions [99], it should also be noted that only 3 of the 18 investigations surpassed the 3-month mark, with 2 additional investigations coming close at the 12-week mark. None of the studies have found an interaction for cfPWV beyond the one investigation with dragon fruit in healthy men and women that noted an interaction at the 3 h mark post-intervention, but this did not persist until the completion of the 14-day study [93]. However, Acanthopanax senticosus Harms fruit extract produced a group-by-time interaction in baPWV in men and women with elevated CVD risk after 12 weeks, but it is surprising that this occurred with the lower dose and not the higher dose [95]. Although only a single acute dose was administered, tomato powder improved bPP among the study population and AIx in a subgroup of individuals for whom this outcome was assessed when compared to the placebo [97]. While none of the trials produced a significant interaction, the results in pomegranate are worth noting. Of the 4 trials that have considered pomegranate, 3 have shown improvements among the treatment group: cfPWV after 28 days [94], PWV after 2 and 4 weeks [96], and bPP after one year [99]. This could suggest potential for pomegranate-based interventions for arterial stiffness that are worth exploring further. Given the small number of studies evaluating these interventions, firm conclusions on whether they have true potential to modulate arterial stiffness are likely premature.

There is strong theoretical rationale for fruit-based interventions to modify the arterial stiffening process. Epidemiological studies have reported significant associations between higher fruit consumption and reduced CVD risk [11,12,13,14], and greater berry and polyphenol intake has been specifically associated with decreased arterial stiffness and associated cBP [15,115]. Because polyphenols possess antioxidative, anti-inflammatory, and anti-nitrosative properties, it is plausible that they may act upon some of the key processes involved in the etiology of arterial stiffness [17]. However, the evidence outlined herein suggests that fruit and fruit-based interventions inconsistently affect the arterial stiffening process, leaving many of these assertions in doubt. Moreover, polyphenols may not be able to directly exert effects on the vascular system through antioxidative capacity due to poor absorption and the inability of the metabolized polyphenols to supply the endogenous antioxidants [116]. Recently, it has been proposed that polyphenols may target the nitric oxide pathway, among others, and that the gut microbiota may regulate the physiological response to polyphenols and lead to divergent individual responses of the cardiovascular system [117]. This may explain some of the heterogeneity of the results found within this review. Furthermore, the gut microbiota of individuals is highly variable and affects the bioavailability of the ingested polyphenols [118]. Nevertheless, polyphenols, their bioactive derivatives, and their capacity to exert health benefits have not been rigorously tested within these intervention studies, and these potential mechanisms are not well understood. Beyond polyphenols, fruit may exert cardioprotective effects through other nutrients such as L-citrulline, L-arginine, potassium, and nitrates, although their specific roles have yet to be established [119]. Additionally, many of the interventions identified for this review were not whole fruits but included juices, extracts, and powders. The bioactive compounds, fiber, and overall nutrient content can differ substantially from their whole fruit counterparts, which can have significant ramifications for bioavailability and potential mechanisms. Therefore, findings from many of these studies may not be extrapolated out to habitual dietary fruit intake.

Caution should be exercised in interpreting these findings from RCTs evaluating fruit-based interventions due to several limitations. Many of the studies contained relatively small sample sizes or did not provide power calculations and, therefore, may have been inadequately powered to detect changes in arterial stiffness. Additionally, no formal quality or risk of bias assessment was performed, although study quality is addressed qualitatively. Another major consideration is whether the intervention durations were sufficient to alter the structure of the arteries that may have resulted from an accumulation of lifestyle choices that spanned decades. Many studies evaluated only acute responses, whereas others may not have been of adequate duration to detect clinically meaningful changes in arterial stiffness. Although there is currently no consensus on the smallest change in cfPWV to result in clinically meaningful change, one recent study suggests that a 1.1 m/s change may be necessary [120], but this has yet to be confirmed. Within the studies found in this review, statistical significance may not equate with clinical significance, and therefore, may not result in measurable changes in cardiovascular risk. In addition, heterogeneity in the assessed arterial stiffness methodologies, populations of interest, and interventions applied complicates comparisons between studies and limits the conclusions that can be drawn. The reliance on multiple surrogate outcomes rather than a single standardized outcome, such as gold-standard cfPWV, further accentuates variability within the reported studies. Moreover, positive findings based on a single study or the inconsistent replication of positive findings diminish confidence in the efficacy of specific fruit-based interventions. When considering the multiple sources of heterogeneity, there is a limited ability to draw causal conclusions within a specified fruit type or across fruit types. Additionally, the diverse interventions, including whole fruits, fruit juices, powders, concentrates, and extracts vary considerably in their composition; thus, the findings cannot be generalized to whole fruit consumption. It also stands to reason that individuals with the highest baseline stiffness have the most to benefit from efficacious interventions, and a lack of change in healthy participants may not truly indicate an inability of the intervention to impact arterial stiffness. Finally, the bioactive compounds from the exogenous food interventions may be metabolized in such a way that bioavailability is diminished.

## 6. Conclusions

Fruit and fruit-derived interventions show potential to improve selected measures of arterial stiffness and central hemodynamics in varied populations, although substantial heterogeneity exists among the fruit-based interventions, populations of interest, arterial stiffness measures evaluated, and study durations. Among the available clinical evidence, double-strength cranberry juice, grapefruit juice, freeze-dried blueberry, and freeze-dried watermelon have demonstrated the most promising findings due to more robust study designs, although confirmation from additional RCTs is warranted. Additional preliminary evidence suggests potential benefits from blackcurrant extract, black raspberry extract, Acanthopanax senticosus Harms fruit extract, tomato powder, and freeze-dried dragon fruit. Nevertheless, more randomized controlled trials with adequate sample sizes, longer intervention durations, and gold-standard measures of arterial stiffness, particularly cfPWV, are necessary to substantiate the current evidence base. Currently, the available evidence is insufficient to support clinically meaningful benefits for most fruits. Furthermore, preclinical and clinical models that can elucidate the mechanisms underlying any improvements in arterial stiffness are also essential. At present, some emerging evidence suggests that the gut microbiota may influence the bioavailability and metabolism of polyphenols found in fruits, which could, in turn, modify the effects on arterial stiffness. Thus, fruit-derived nutraceuticals may be a natural and non-pharmacological approach to address the stiffening of arteries that occurs with aging, obesity, and various disease states, but more high-quality studies are needed before their effectiveness can be established.

## 7. Future Directions

Additional large-scale clinical trials with longer durations are warranted, likely with durations that exceed the 3-month mark. Still, the duration for nutritional interventions, especially those that do not include weight loss or significant changes in dietary patterns, to produce meaningful effects on arterial stiffness is unknown and requires further investigation. Many fruit-based interventions have yet to be studied and even the most promising findings have yet to be replicated across multiple investigations, leaving many opportunities for research in this area. When feasible, interventions should be applied among individuals with higher baseline stiffness to assess efficacy. Furthermore, studies that can elucidate the bioavailability of different formulations and mechanisms by which polyphenols or other bioactive compounds can exert their effects are needed.

## Figures and Tables

**Table 1:** Clinical trials considering blueberries and blueberry-based interventions for arterial stiffness.

Intervention	Location	Population of Interest	Treatment Used	Subjects Enrolled (Subjects Completed)	Treatment Duration	Arterial Stiffness Measures Evaluated	Primary Findings	Reference
Freeze-dried blueberry powder	United Kingdom	Healthy older men and women	Treatment: 26 g of freeze-dried blueberry powder Control: 26 g of placebo powder	Treatment: 35 (27) Control: 31 (27)	12 weeks	cfPWV, AIx@HR75	No significant changes or interaction	Wood et al., 2023 [49]
Blueberry mousse	Italy	Healthy older men and women	Treatment: 250 g blueberry mousse Control: 250 mL of sugar water	Treatment: 20 (16) Control: 20 (16)	Single acute dose	AIx or AIx@HR75	No significant changes or interaction	Tucci et al., 2024 [50]
Freeze-dried blueberry powder	USA	Postmenopausal women with elevated BP	Treatment: 22 g freeze-dried blueberry powder Control: 22 g control powder	Treatment: 25 (20) Control: 23 (20)	8 weeks	cfPWV, baPWV	Significant group-by-time interaction in baPWV	Johnson et al., 2015 [43]
Fresh blueberry and freeze-dried blueberry powder	United Kingdom	Generally healthy men and women	Treatment I: 160 g of fresh whole blueberry Treatment II: 20 g of freeze-dried blueberry powder Control: placebo capsule	Treatment I: 42 (37) Treatment II: 42 (37) Control: 42 (37)	1 week	crPWV	No significant changes or interaction	Wang et al., 2022 [45]
Frozen thawed blueberry	Italy	Healthy smoking and non-smoking young men	Treatment I: 300 g of thawed frozen blueberry Control: control beverage	Treatment: 24 (24) Control: 24 (24)	Single acute dose	AIx or AIx@HR75	No significant changes or interaction	Del Bo’ et al., 2017 [51]
Freeze-dried blueberry powder	USA	Postmenopausal women with elevated BP	Treatment I: 22 g freeze-dried blueberry powder Control: 22 g placebo powder	Treatment: 24 (22) Control: 24 (21)	12 weeks	cfPWV, AIx, AIx@HR75, cBP	cPP was significantly reduced in the treatment group at 4 weeks compared to baseline, but not at 8 or 12 weeks	Woolf et al., 2023 [48]
Freeze-dried blueberry powder	USA	Men and postmenopausal women	Treatment: 38 g freeze-dried blueberry powder Control: placebo powder	Treatment: 13 (13) Control: 12 (12)	6 weeks	cfPWV, AIx, cBP	Reduction in AIx and cSBP in the treatment group compared to baseline	McAnulty et al., 2014 [47]
Freeze-dried blueberry powder	United Kingdom	Overweight and obese adults with MetS	Treatment I: 26 g freeze-dried blueberries Treatment II: 13 g freeze-dried blueberries, 13 g placebo powder Control: 26 g placebo powder	Treatment I: 44 (37) Treatment II: 49 (39) Control: 45 (39)	6 months	cfPWV, AIx@HR75	AIx@HR75 significantly reduced in treatment I compared to treatment II and control groups	Curtis et al., 2019 [46]
Frozen thawed blueberry	Italy	Smoking young men	Treatment I: 300 g fresh-frozen blueberries + smoking Treatment II: smoking one cigarette Control: 300 mL sugar water + smoking	Treatment I: 16 (16) Treatment II: 16 (16) Control: 16 (16)	Single acute dose	AIx, AIx@HR75	A reduction in AIx occurred after Treatment II but not Treatment I	Del Bo’ et al., 2014 [52]
Freeze-dried blueberry powder	USA	Middle-aged and older men and women	Treatment: 38 g freeze-dried blueberry powder Control: placebo powder	Treatment: 10 (10) Control: 12 (12)	3 weeks	cfPWV, AIx	Significant time effects for AIx	McAnulty et al., 2019 [53]
Freeze-dried blueberry powder *	Italy	Men and women (details NR)	Treatment: 30 g of freeze-dried blueberry powder Control: placebo drink	Treatment: 20 (NR) Control: 20 (NR)	Single acute dose	AIx	Significant time effects for AIx in a preliminary analysis of first 12 subjects	Rendine et al., 2024 [54]
Blueberry drink from freeze-dried blueberry powder	United Kingdom	Healthy young men	Treatment I: blueberry drink with 34 g freeze-dried blueberry powder Treatment II: blueberry drink with 57 g freeze-dried blueberry powder Treatment III: blueberry drink with 80 g freeze-dried blueberry powder Control: control drink	Treatment I: 11 (10) Treatment II: 11 (10) Treatment III: 11 (10) Control: 11 (10)	Single acute dose	cfPWV, AIx, DVP-SI, DVP-RI, cBP	No significant changes or interaction	Rodriguez-Mateos et al., 2013 [55]
Blueberry juice	China	Young men and women	Treatment I: low-intensity aerobic exercise Treatment II: low-volume (25 mL) blueberry juice Treatment III: mid-volume (50 mL) blueberry juice Treatment IV: high-volume (100 mL) blueberry juice Treatment V: low-intensity aerobic exercise + low-volume (25 mL) blueberry juice Treatment VI: low-intensity aerobic exercise + mid-volume (50 mL) blueberry juice Treatment VII: low-intensity aerobic exercise + high-volume (100 mL) blueberry juice Control: No exercise or nutrition intervention	Treatment I: 6 (6) Treatment II: 6 (6) Treatment III: 6 (6) Treatment IV: 6 (6) Treatment V: 6 (6) Treatment VI: 6 (6) Treatment VII: 6 (6) Control: 6 (6)	Single acute dose	baPWV	Significant main effect for time in baPWV	Wu et al., 2025 [44]

* Conference abstract only; AIx = augmentation index; AIx@HR75 = augmentation index standardized to a heart rate of 75 beats per minute; baPWV = brachial-ankle pulse wave velocity; BP = blood pressure; cBP = central blood pressure; cfPWV = carotid-femoral pulse wave velocity; crPWV = carotid-radial pulse wave velocity; cPP = central pulse pressure; cSBP = central systolic blood pressure; DVP-RI = digital volume pulse reflection index; DVP-SI = digital volume pulse stiffness index; MetS = metabolic syndrome; NR = not reported.

**Table 2:** Clinical trials considering aronia berries, blackberries, blackcurrants, cranberries, raspberries, strawberries, mixed berries, and berry-based interventions for arterial stiffness.

**Intervention**	**Location**	Population of Interest	Treatment Used	Subjects Enrolled (Subjects Completed)	Treatment Duration	Arterial Stiffness Measures Evaluated	Primary Findings	Reference
Aronox^®^ aronia berry extract	United Kingdom	Middle-aged and older men and women with elevated BP	Treatment: 500 mg Aronox^®^ aronia berry extract capsules Control: placebo capsules	Treatment: 51 (45) Control: 51 (48)	12 weeks	Awake PWV, 24 h brachial AIx, 24 h aortic AIx, awake brachial AIx, and awake aortic AIx, cBP	Awake PWV, 24 h brachial AIx, 24 h aortic AIx, awake brachial AIx, and awake aortic AIx were improved in treatment group after 12 weeks in a group-by-time interaction, but this change did not persist ~24 h after the last capsule was taken	Le Sayec et al., 2022 [59]
Concentrated aronia berry extract and aronia berry whole fruit capsules	United Kingdom	Healthy men	Treatment I: 500 mg capsules made from 75 g aronia berry minus fiber and organic acids Treatment II: 500 mg capsules made from 10 g aronia whole fruit Control: 500 mg placebo capsule	Treatment I: 23 (23) Treatment II: 23 (23) Control: 20 (20)	12 weeks + single acute dose	cfPWV, AIx, cBP	No significant changes or interaction	Istas et al., 2019 [64]
Unripe black raspberry extract	South Korea	Men and women with MetS	Treatment: 750 mg black raspberry powder Control: placebo capsules	Treatment: 26 (25) Control: 25 (25)	12 weeks	AIx, cBP	The change in AIx for the treatment was significantly different than the control group	Jeong et al., 2016 [61]
Blackberry polyphenol-enriched beverage *	Republic of Ireland	Men and women with untreated elevated BP	Treatment: 250 mL blackberry polyphenol-enriched beverage Control: 250 mL low-dose polyphenol beverage	Treatment: 83 (80) Control: 83 (80)	6 weeks	cBP	No significant changes or interaction	Heneghan et al., 2017 [65]
CurraNZ^®^ blackcurrant extract	Japan	Older men and women	Treatment: 600 mg CurraNZ^®^ blackcurrant extract capsules Control: 600 mg placebo capsule	Treatment: 14 (14) Control: 14 (14)	7 days	cfPWV, faPWV, AIx, cBP	cfPWV and cSBP decreased significantly in the treatment group relative to the placebo group in a group-by-time interaction; AIx decreased significantly in the treatment group, but no interaction occurred	Okamoto et al., 2020 [58]
Freeze-dried cranberry powder	Germany	Healthy men	Treatment: 9 g freeze-dried cranberry powder Control: 9 g placebo powder	Treatment: 23 (22) Control: 22 (22)	1 month	cfPWV, AIx	No significant changes or interaction	Heiss et al., 2022 [66]
Double-strength cranberry juice	USA	Men and women with stable coronary artery disease	Treatment: 480 mL double-strength cranberry juice Control: 480 mL placebo beverage	Treatment: 47 (44) Control: 47 (44)	4 weeks	cfPWV, crPWV	cfPWV was decreased by 0.5 m/s in the treatment group in a group-by-time interaction	Dohadwala et al., 2011 [57]
Cranberry juice with differing amounts of total polyphenols	Germany	Healthy young men	Treatment I: 450 mL cranberry juice (409 mg total polyphenols) Treatment II: 450 mL cranberry juice (787 mg total polyphenols) Treatment III: 450 mL cranberry juice (1238 mg total polyphenols) Treatment IV: 450 mL cranberry juice (1534 mg total polyphenols) Treatment V: 450 mL cranberry juice (1910 mg total polyphenols) Control: control drink (3 mg total polyphenols)	Treatment I: 10 (10) Treatment II: 10 (10) Treatment III: 10 (10) Treatment IV: 10 (10) Treatment V: 10 (10) Control: 10 (10)	Single acute dose	cfPWV, AIx, cBP	A significant decrease in AIx from baseline was observed at 1.5 and 6 h for Treatment IV and at 4 and 6 h for Treatment I; a significant decrease in cSBP from baseline was observed at 6 h for Treatment V compared to baseline	Rodriguez-Mateos et al., 2016 [62]
Cranberry juice	USA	Men and women with elevated BP	Treatment: 500 mL cranberry juice Control: 500 mL placebo juice	Treatment: 47 (40) Control: 47 (40)	8 weeks	cfPWV, AIx, cBP	No significant changes or interaction	Richter et al., 2021 [67]
Frozen red raspberry beverage	Germany	Healthy young men	Treatment I: 592 mL beverage (400 g red raspberries) Treatment II: 592 mL beverage (200 g red raspberries) Control: 592 mL placebo drink	Treatment I: 10 (10) Treatment II: 10 (10) Control: 10 (10)	Single acute dose	cfPWV, AIx, cBP	No significant changes or interaction	Istas et al., 2018 [68]
Mixed red berry fruit juice	Germany	Pre-diabetic men and women	Treatment: 200 mL red berry fruit juice Control: 200 mL placebo drink	Treatment: 13 (10) Control: 13 (10)	14 days	PWV	Significant time effects resulted for PWV	Valder et al., 2025 [63]
Freeze-dried strawberry powder	USA	Overweight and obese men and women	Treatment I: 40 g “high dose” freeze-dried strawberry powder Treatment II: 40 g “low dose” freeze-dried strawberry powder Control: 40 g placebo powder	Treatment: 42 (40) Control: 42 (40)	4 weeks	PWV, AIx, cBP	No significant changes or interaction	Richter et al., 2023 [69]
Freeze-dried strawberry powder	USA	Postmenopausal women with elevated BP	Treatment I: 50 g freeze-dried strawberry powder Treatment II: 25 g freeze-dried strawberry powder Control: 25 g placebo powder	Treatment I: 24 (20) Treatment II: 25 (20) Control: 22 (20)	8 weeks	baPWV, faPWV	baPWV and faPWV were reduced from baseline in treatment II group	Feresin et al., 2017 [60]

* Conference abstract only; AIx = augmentation index; baPWV = brachial-ankle pulse wave velocity; BP = blood pressure; cBP = central blood pressure; cfPWV = carotid-femoral pulse wave velocity; crPWV = carotid-radial pulse wave velocity; cSBP = central systolic blood pressure; faPWV = femoral-ankle pulse wave velocity; GAE = gallic acid equivalents; MetS = metabolic syndrome; NR = not reported; PWV = pulse wave velocity.

**Table 3:** Clinical trials considering tart cherries and tart cherry-based interventions for arterial stiffness.

Intervention	Location	Population of Interest	Treatment Used	Subjects Enrolled (Subjects Completed)	Treatment Duration	Arterial Stiffness Measures Evaluated	Effects on Arterial Stiffness	Reference
CherryActive^®^ Montmorency tart cherry concentrate	United Kingdom	Men with early hypertension	Treatment: 60 mL CherryActive^®^ Montmorency tart cherry concentrate Control: 60 mL fruit-flavored cordial	Treatment: 16 (15) Control: 16 (15)	Single acute dose	cfPWV, AIx, AIx@HR75, DVP-RI, DVP-SI	A time effect was present for cfPWV and DVP-RI	Keane et al., 2016 [70]
Montmorency tart cherry juice	USA	Men and women with MetS	Treatment: 480 mL tart cherry juice Control: 480 mL placebo drink	Treatment: 13 (9) Control: 13 (10)	12 weeks	cfPWV, baPWV, faPWV, AIx, AIx@HR75, cBP, AP	No significant changes or interaction	Johnson et al., 2020 [74]
Montmorency tart cherry	United Kingdom	Men and women at risk for type 2 diabetes	Treatment: 60 mL of Montmorency tart cherry Control: 60 mL of Kool-aid	Treatment: 28 (25) Control: 28 (25)	3 months	cfPWV, AIx, AIx@HR75	No significant changes or interaction	Kimble et al., 2021 [75]
CherryActive^®^ Montmorency tart cherry concentrate	United Kingdom	Healthy men and women	Treatment: 60 mL CherryActive^®^ Montmorency tart cherry concentrate Control: 260 mL black cherry cordial	Treatment: NR (12) Control: NR (11)	4 weeks	PWV, DVP-SI, DVP-RI	No significant changes or interaction	Kimble et al., 2021 [76]
CherryActive^®^ Montmorency tart cherry concentrate or capsules	United Kingdom	Men and women with MetS	Treatment I: 30 mL Montmorency tart cherry concentrate Treatment II: 10 capsules of freeze-dried Montmorency tart cherry skin powder Control: 30 mL fruit-flavored cordial	Treatment: 13 (11) Control: 13 (11)	Single acute dose	AIx, AIx@HR75, cBP, cPP, AP	Main effect for time in AIx, AIx@HR75, and AP	Desai et al., 2019 [72]
CherryActive^®^ Montmorency tart cherry concentrate	United Kingdom	Men and women with MetS	Treatment I: 30 mL Montmorency tart cherry concentrate Control: 30 mL fruit-flavored cordial	Treatment: 15 (12) Control: 15 (12)	7 days	AIx, AIx@HR75, cBP, bPP, AP	An acute main effect of condition was found for AIx@HR75	Desai et al., 2021 [71]
CherryActive^®^ Montmorency tart cherry concentrate	United Kingdom	Healthy men and women	Treatment: 30 mL Cherry Active^®^ cherry juice concentrate Control: 250 mL Sprite^®^ control drink	Treatment: 26 (25) Control: 21 (21)	6 weeks	bkPWV	No significant changes or interaction	Lynn et al., 2014 [31]
CherryActive^®^ Montmorency tart cherry concentrate	United Kingdom	Healthy men and women	Treatment: 30 mL Cherry Active^®^ cherry juice concentrate Control: 250 mL low-nitrate water	Treatment: 13 (12) Control: 13 (12)	Single acute dose	cfPWV, AIx, cBP	A main effect for time was detected for cSBP and cDBP	Lamb et al., 2026 [73]

AIx = augmentation index; AIx@HR75 = augmentation index standardized to a heart rate of 75 beats per minute; AP = augmentation pressure; baPWV = brachial-ankle pulse wave velocity; bkPWV = brachial-knee pulse wave velocity; cBP = central blood pressure; cDBP = central diastolic blood pressure; cfPWV = carotid-femoral pulse wave velocity; cPP = central pulse pressure; cSBP = central systolic blood pressure; DVP-RI = digital volume pulse reflection index; DVP-SI = digital volume pulse stiffness index; faPWV = femoral-ankle pulse wave velocity; NR = not reported; PWV = pulse wave velocity.

**Table 4:** Clinical trials considering grape-based interventions for arterial stiffness.

Intervention	Location	Population of Interest	Treatment Used	Subjects Enrolled (Subjects Completed)	Treatment Duration	Arterial Stiffness Measures Evaluated	Effects on Arterial Stiffness	Reference
Welch’s Concord grape juice	Greece	Men and women smokers	Treatment: 7 cc/kg of Welch’s Concord grape juice Control: 7 cc/kg of grapefruit juice	Treatment: 26 (NR) Control: 26 (NR)	14 days	cfPWV	Reduction in cfPWV in the treatment group compared to baseline; treatment also prevented the acute smoking-induced increase in cfPWV at days 7 and 14	Siasos et al., 2014 [77]
Welch’s Concord grape juice	USA	Men and women with elevated BP	Treatment: 7 mL/kg Concord grape juice Control: 7 mL/kg placebo beverage	Treatment: 83 (64) Control: 83 (64)	8 weeks	cfPWV, crPWV, bPP	No significant changes or interaction	Dohadwala et al., 2010 [79]
Meganatural^®^-BP grape seed extract	USA	Young men	Treatment: 300 mg Meganatural^®^ grape seed extract Control: 300 mg starch	Treatment: 10 (NR) Control: 10 (NR)	Single acute dose	Aortic stiffness by bioelectrical impedance	No significant changes or interaction	Dillon et al., 2020 [80]
Gravinol™ grape seed proanthocyanidin extract	Japan	Prehypertensive men and women	Treatment I: 200 mg Gravinol™ “low dose” grape seed extract Treatment II: 400 mg Gravinol™ “high dose” grape seed extract Control: 0 mg grape seed extract placebo	Treatment I: 10 (10) Treatment II: 10 (10) Control: 10 (10)	12 weeks	PWV	PWV improved with Treatment II at the 8- and 12-week marks compared to baseline	Odai et al., 2019 [78]
Grape seed polyphenols	Australia	Hypertensive men and women	Treatment I: 500 mg/d of vitamin C and matched grape seed polyphenols placebo Treatment II: 1000 mg/d of grape seed polyphenols and matched vitamin C placebo Treatment III: 500 mg/d of vitamin C and 1000 mg/d of grape seed polyphenols Control: matched placebo tablets for both grape seed polyphenols and vitamin C	Treatment I: 19 (19) Treatment II: 18 (16) Treatment III: 18 (16) Control: 19 (18)	6 weeks	bPP	No significant differences occurred with Treatment II alone, but Treatment III resulted in a higher rate of nighttime bPP variation (detrimental effect)	Hodgson et al., 2014 [81]
Raisins	Greece	Men and women smokers	Treatment: normal smoking habits + 90 g raisins Control: normal smoking habits	Treatment: 22 (20) Control: 14 (13)	4 weeks	cfPWV, crPWV	No significant changes or interaction	Kanellos et al., 2017 [82]

bPP = brachial pulse pressure; cfPWV = carotid-femoral pulse wave velocity; crPWV = carotid-radial pulse wave velocity; GAE = gallic acid equivalents; NR = not reported; PWV = pulse wave velocity.

**Table 5:** Clinical trials considering citrus-based interventions for arterial stiffness.

Intervention	Location	Population of Interest	Treatment Used	Subjects Enrolled (Subjects Completed)	Treatment Duration	Arterial Stiffness Measures Evaluated	Primary Findings	Reference
Blood orange juice	United Kingdom	Men and women	Treatment: 500 mL blood orange juice Control: 500 mL standard orange juice	Treatment: 45 (41) Control: 45 (41)	28 days	cfPWV, baPWV, cBP	No significant changes or interaction	Hollands et al., 2018 [87]
Grapefruit juice	France	Postmenopausal women	Treatment: 340 mL grapefruit juice Control: 340 mL control drink	Treatment: 52 (48) Control: 52 (48)	6 months	cfPWV, AIx@HR75	cfPWV was lower in the treatment group compared to placebo in a group-by-time interaction when adjusted for baseline values	Habauzit et al., 2015 [83]
Concentrated β-cryptoxanthin-rich Satsuma mandarin juice	Japan	Men and women	Treatment: 125 mL concentrated β-cryptoxanthin-rich Satsuma mandarin juice Control: 125 mL β-cryptoxanthin-deprived Satsuma mandarin juice	Treatment: 59 (50) Control: 59 (50)	12 weeks	baPWV	baPWV had increased in both the treatment and control groups 8 months post-intervention, but a significant group-by-time interaction in baPWV occurred, revealing a significantly lower baPWV in the treatment group	Nakamura et al., 2017 [84]
Orange juice and hesperidin-enriched orange juice	Spain	Men and women with elevated BP	Treatment I: 500 mL orange juice Treatment II: 500 mL hesperidin-enriched orange juice Control: 500 mL control drink	Treatment I: 53 (46) Treatment II: 53 (40) Control: 53 (43)	12 weeks	bPP	bPP decreased in a dose-dependent manner relative to the hesperidin content of the treatment beverages at 2 weeks, 6 weeks, and 10 weeks, but the linear trend did not reach significance at 12 weeks; bPP was significantly reduced at 2 h, 4 h, and 6 h after a single acute dose of treatment II only	Valls et al., 2021 [85]
Orange juice and hesperidin supplement	United Kingdom	Healthy older men	Treatment I: hesperidin supplement Treatment II: 767 mL orange juice Control: 767 mL placebo drink	Treatment: 16 (14) Control: 16 (14)	Single acute dose	cfPWV, AIx@HR75	No significant changes or interaction	Schär et al., 2015 [86]

AIx@HR75 = augmentation index standardized to a heart rate of 75 beats per minute; baPWV = brachial-ankle pulse wave velocity; BP = blood pressure; bPP = brachial pulse pressure; cBP = central blood pressure; cfPWV = carotid-femoral pulse wave velocity; NR = not reported.

**Table 6:** Clinical trials considering watermelon-based interventions for arterial stiffness.

Intervention	Location	Population of Interest	Treatment Used	Subjects Enrolled (Subjects Completed)	Treatment Duration	Arterial Stiffness Measures Evaluated	Primary Findings	Reference
Freeze-dried watermelon extract	USA	Postmenopausal women	Treatment: 6 g freeze-dried watermelon extract Control: placebo powder	Treatment: 12 (12) Control: 12 (12)	6 weeks	baPWV, AIx, AIx@HR75	baPWV, cSBP, and cDBP decreased significantly in the treatment group in a group-by-time interaction	Figueroa et al., 2013 [88]
Wild watermelon juice	Japan	Young, healthy women	Treatment: 100 mL watermelon juice Control: 100 mL placebo beverage	Treatment: 12 (NR) Control: 12 (NR)	Single acute dose	cfPWV, baPWV, faPWV	A significant interaction in the percentage change in faPWV, with a significant reduction at 30 min, but no further changes at 60 and 90 min	Fujie et al., 2022 [89]
Freeze-dried watermelon extract	USA	Prehypertensive men and women	Treatment: 4 g freeze-dried watermelon powder Control: placebo powder	Treatment: 9 (9) Control: 9 (9)	6 weeks	cfPWV, AIx, AIx@HR75, bPP, cBP	The difference between the treatment and control groups in AIx, AIx@HR75, bPP, cPP, cSBP was significantly larger at 6 weeks than baseline	Figueroa et al., 2011 [91]
Freeze-dried watermelon extract	USA	Obese men and women with elevated BP	Treatment: 6 g freeze-dried watermelon powder Control: placebo powder	Treatment: 14 (14) Control: 14 (14)	5 weeks	AIx	Significant reduction in AIx in a group-by-time interaction	Figueroa et al., 2012 [90]
Watermelon juice	USA	Postmenopausal women	Treatment: 720 mL watermelon juice Control: placebo drink	Treatment: 21 (17) Control: 21 (17)	4 weeks	PWV, bPP	No significant changes or interaction	Ellis et al., 2021 [92]

AIx = augmentation index; AIx@HR75 = augmentation index standardized to a heart rate of 75 beats per minute; baPWV = brachial-ankle pulse wave velocity; BP = blood pressure; bPP = brachial pulse pressure; cDBP = central diastolic blood pressure; cfPWV = carotid-femoral pulse wave velocity; cPP = central pulse pressure; cSBP = central systolic blood pressure; faPWV = femoral-ankle pulse wave velocity; NR = not reported; PWV = pulse wave velocity.

**Table 7:** Clinical trials considering other fruit and fruit-based interventions for arterial stiffness.

Intervention	Location	Population of Interest	Treatment Used	Subjects Enrolled (Subjects Completed)	Treatment Duration	Arterial Stiffness Measures Evaluated	Primary Findings	Reference
Apple flavanol extract	United Kingdom	Older men and women with elevated BP	Treatment I: single dose apple flavanol extract Treatment II: double dose apple flavanol extract Treatment III: apple procyanidin extract Control: microcrystalline cellulose	Treatment I: 43 (42) Treatment II: 43 (42) Treatment III: 43 (42) Control: 43 (42)	Single acute dose + 4 weeks	cfPWV, baPWV, AIx, cBP	No significant changes or interaction	Hollands et al., 2018 [100]
Fresh skins of pink apples	Australia	Men and women at risk for CVD	Treatment: one whole Cripps Pink apple with skin + skin only from a second Cripps Pink apple Control: flesh only of one Cripps pink apple	Treatment: 36 (30) Control: 36 (30)	Single acute dose + 4 weeks	PWV, AIX@HR75, cBP	No significant changes or interaction	Bondonno et al., 2018 [101]
Fresh apples	United Kingdom	Generally healthy men and women	Treatment: 340 g serving of apples, or 2 apples Control: 500 mL apple juice concentrate	Treatment: 43 (40) Control: 43 (40)	8 weeks	AIX@HR75, cBP	No significant changes or interaction	Koutsos et al., 2020 [102]
Fresh avocado	USA	Obese men and women	Treatment: 1 avocado + habitual diet Control: habitual diet (limited to 2 or less avocados per month)	Treatment: 67 (65) Control: 67 (64)	6 months	cfPWV, AIx, cBP	No significant changes or interaction	Davis et al., 2024 [103]
Fresh avocado and mango	USA	Overweight and obese men and women	Treatment: partially controlled diet reflecting the typical American diet + 1 Hass avocado + 1 cup mango daily Control: partially controlled diet reflecting the typical American diet + equicaloric carbohydrate food alternatives for the avocado and mango	Treatment: 41 (30) Control: 41 (38)	8 weeks	cfPWV, cBP	No significant changes or interaction	Preiss et al., 2026 [104]
Freeze-dried dragon fruit	United Kingdom	Healthy men and women	Treatment: 24 g freeze-dried red dragon fruit pulp powder Control: 24 g placebo powder	Treatment (acute): 20 (19) Control (acute): 20 (19) Treatment: 20 (18) Control: 20 (18)	Single acute dose + 14 days	cfPWV, AIx@HR75, cBP	Significant decrease in cfPWV of 0.5 m/s at 3 h post-consumption compared to placebo; significant 7% reduction in AIx@HR75 in the treatment group relative to the placebo group at 14 days	Cheok et al., 2022 [93]
Acanthopanax senticosus Harms fruit extract	South Korea	Men and women at risk for CVD	Treatment I: 1000 mg Acanthopanax senticosus Harms fruit (“high dose”) capsule Treatment II: 500 mg Acanthopanax senticosus Harms fruit (“low dose”) capsule Control: placebo capsule	Treatment I: 34 (25) Treatment II: 33 (26) Control: 31 (25)	12 weeks	baPWV	Significant group-by-time interaction showing a reduction in baPWV with Treatment II relative to the placebo group	Oh et al., 2020 [95]
Mixed dried fruit	USA	Overweight and obese men and women at risk for CVD	Treatment: 112 g of dried fruit consisting of 28 g dried plums, 28 g, Black Mission figs, 28 g Deglet Noor dates, and 28 g raisins Control: 43 g animal crackers + 51 g fruit snack gummies	Treatment: 55 (52) Control: 55 (52)	4 weeks	cfPWV, AIx, AP, cBP	cDBP increased for the control condition only	Sullivan et al., 2020 [105]
Proliva^®^ olive fruit extract	Spain	Men and women	Treatment I: 250 mg Proliva^®^ standard olive fruit extract Treatment II: 500 mg Proliva ^®^ standard olive fruit extract Control: contents NR	Treatment I: 12 (NR) Treatment II: 12 (NR) Control: 12 (NR)	11 days	CAVI	CAVI scores were reduced in treatment and control groups with no differences between groups	Pais et al., 2016 [106]
Fresh pear	USA	Men and women with MetS	Treatment: 2 medium sized fresh pears daily Control: 50 g of calorie-matched control drink powder	Treatment: 50 (40) Control: 50 (40)	12 weeks	bPP	bPP was significantly reduced in treatment group at 6 and 12 weeks compared to baseline	Navaei et al., 2019 [98]
Pomegranate juice	Israel	Men and women on chronic dialysis	Treatment: 100 mL pomegranate juice Control: 100 mL placebo juice	Treatment: 66 (41) Control: 35 (26)	One year	bPP	bPP was significantly reduced in the treatment group from baseline by 8.8%	Shema-Didi et al., 2014 [99]
Pomegranate Pomanox^®^ P30 extract	United Kingdom	Men and women	Treatment: 1.08 g Pomanox^®^ P30 pomegranate fruit extract capsule Control: 1.08 g placebo capsule	Treatment: 12 (12) Control: 12 (12)	28 days	cfPWV	cfPWV was significantly reduced in the treatment group from baseline	Al-Dujaili et al., 2022 [94]
Pomegranate juice	United Kingdom	Healthy men and women	Treatment: 330 mL pomegranate juice Control: 330 mL lemonade	Treatment: 26 (24) Control: 25 (24)	4 weeks	PWV	No significant changes or interaction	Lynn et al., 2012 [107]
Pomegranate juice	United Kingdom	Overweight and obese men and women	Treatment: 500 mL pomegranate juice Control: 500 placebo drink	Treatment: NR (28) Control: NR (28)	4 weeks	PWV	PWV was significantly reduced in the treatment group at 2 weeks and 4 weeks compared to baseline	Tsang et al., 2012 [96]
Dried plums (prunes)	USA	Healthy postmenopausal women	Treatment: 6 dried plums (prunes) Control: 2 dried prunes	Treatment: 27 (27) Control: 27 (27)	2 weeks	AIx, AIx@HR75	No significant changes or interaction	Al-Dashti et al., 2019 [108]
Fruitflow^®^ tomato powder	Norway	Apparently healthy men	Treatment: 150 mg Fruitflow^®^ powder Control: placebo powder	Treatment: 18 (12) Control: 18 (12)	Single acute dose	bPP, AIx	bPP was lower in the treatment group over 24 h time course than the placebo; AIx tested in a subgroup (*n* = 6) was lowered by 8% in the treatment group compared to placebo over 24 h time course	Uddin et al., 2018 [97]
Tomato-based foods	United Kingdom	Men and women	Treatment I: Diet low in tomato-based foods + 10 mg lycopene Treatment II: High tomato-based products Control: Diet low in tomato-based foods	Treatment I: 79 (68) Treatment II: 84 (81) Control: 81 (76)	16 weeks	PWV	No significant changes or interaction	Thies et al., 2012 [109]
Tomato paste *	Portugal	Young, sedentary men and women	Treatment: 70 g tomato paste Control: contents NR	Treatment: 30 (NR) Control: 30 (NR)	4 weeks	cfPWV, AIx, cBP	No significant changes or interaction	Pereira et al., 2015 [110]

* Conference abstract only; AIx = augmentation index; AIx@HR75 = augmentation index standardized to a heart rate of 75 beats per minute; AP = augmentation pressure; baPWV = brachial-ankle pulse wave velocity; BP = blood pressure; bPP = brachial pulse pressure; CAVI = cardio-ankle vascular index; cBP = central blood pressure; cDBP = central diastolic blood pressure; cfPWV = carotid-femoral pulse wave velocity; cSBP = central systolic blood pressure; CVD = cardiovascular disease; DVP-RI = digital volume pulse reflection index; DVP-SI = digital volume pulse stiffness index; GAE = gallic acid equivalents; NR = not reported; PWV = pulse wave velocity.

**Table 8:** Summary of the main bioactive compounds, reported effects on vascular outcomes, and strength of evidence for the different fruit categories.

Fruit Category	Main Bioactive Compound(s)	Improvements in Gold-Standard cfPWV	Improvements in Other PWV Measures	Improvements in Central Hemodynamics	Strength of Evidence
Blueberries	Anthocyanins	−	+	+	Moderate
Other Berries	Anthocyanins, Tannins, Proanthocyanidins, Flavonols	+	+	+	Limited
Tart Cherries	Anthocyanins	−	−	−	Insufficient
Grapes	Proanthocyanidins, Anthocyanins, Resveratrol	+	+	−	Limited
Citrus	Flavonoids	+	-	−	Moderate
Watermelon	L-Citrulline	−	+	+	Moderate
Other Fruits	Mixed	+	−	−	Limited

## Data Availability

No new data were created or analyzed in this study. Data sharing is not applicable to this article.

## Supplementary Materials

The following supporting information can be downloaded at: https://www.mdpi.com/article/10.3390/nu18162627/s1.
