# Supplementary material for: The Modulating Effects of Fruit and Fruit-Derived Interventions on Arterial Stiffness
Source: Nutrients. 2026 Aug 12;18(16):2627. doi: 10.3390/nu18162627 (PMC13516526; doi:10.3390/nu18162627)
Supplement: Supplementary file 1 [file nutrients-18-02627-s001.zip › nutrients-4468377-supplementary.pdf]

SEARCH STRATEGY

PubMed

Search: ("arterial stiffness" OR "aortic stiffness" OR "cfPWV" OR "PWV" OR “pulse pressure” OR “wave reflections” OR “augmentation index” OR “pulse wave velocity” OR “central pressure” OR “arterial compliance”) AND (“fruit” OR “berry” OR “apple” OR “blueberry” OR “grape” OR “watermelon” OR “berries” OR “orange” OR “pomegranate” OR “grapefruit” OR “cherry” OR “tomato” OR “avocado” OR “currant” OR “cranberry” OR “strawberry” OR “elderberry” OR “raspberry” OR “bilberry” OR “blackberry” OR “pineapple” OR “olive” OR “pear” OR “plum”)

Filter: “Clinical Trials”

Embase

Search: ("arterial stiffness" OR "aortic stiffness" OR "cfPWV" OR "PWV" OR “pulse pressure” OR “wave reflections” OR “augmentation index” OR “pulse wave velocity” OR “central pressure” OR “arterial compliance”) AND “fruit” OR “berry” OR “apple” OR “blueberry” OR “grape” OR “watermelon” OR “berries” OR “orange” OR “pomegranate” OR “grapefruit” OR “cherry” OR “tomato” OR “avocado” OR “currant” OR “cranberry” OR “strawberry” OR “elderberry” OR “raspberry” OR “bilberry” OR “blackberry” OR “pineapple” OR “olive” OR “pear” OR “plum”)

Sources: “Clinical Trials”

Cochrane Library

Search: ("arterial stiffness" OR "aortic stiffness" OR "cfPWV" OR "PWV" OR “pulse pressure” OR “wave reflections” OR “augmentation index” OR “pulse wave velocity” OR “central pressure” OR “arterial compliance”) AND “fruit” OR “berry” OR “apple” OR “blueberry” OR “grape” OR “watermelon” OR “berries” OR “orange” OR “pomegranate” OR “grapefruit” OR “cherry” OR “tomato” OR “avocado” OR “currant” OR “cranberry” OR “strawberry” OR “elderberry” OR “raspberry” OR “bilberry” OR “blackberry” OR “pineapple” OR “olive” OR “pear” OR “plum”)

Web of Science

Search: ("arterial stiffness" OR "aortic stiffness" OR "cfPWV" OR "PWV" OR “pulse pressure” OR “wave reflections” OR “augmentation index” OR “pulse wave velocity” OR “central

pressure” OR “arterial compliance”) AND (“fruit” OR “berry” OR “apple” OR “blueberry” OR “grape” OR “watermelon” OR “berries” OR “orange” OR “pomegranate” OR “grapefruit” OR “cherry” OR “tomato” OR “avocado” OR “currant” OR “cranberry” OR “strawberry” OR “elderberry” OR “raspberry” OR “bilberry” OR “blackberry” OR “pineapple” OR “olive” OR “pear” OR “plum”)

CINAHL

Search: ("arterial stiffness" OR "aortic stiffness" OR "cfPWV" OR "PWV" OR “pulse pressure” OR “wave reflections” OR “augmentation index” OR “pulse wave velocity” OR “central pressure” OR “arterial compliance”) AND (“fruit” OR “berry” OR “apple” OR “blueberry” OR “grape” OR “watermelon” OR “berries” OR “orange” OR “pomegranate” OR “grapefruit” OR “cherry” OR “tomato” OR “avocado” OR “currant” OR “cranberry” OR “strawberry” OR “elderberry” OR “raspberry” OR “bilberry” OR “blackberry” OR “pineapple” OR “olive” OR “pear” OR “plum”)

Filter: “Academic journals”

Table S1: Clinical trials considering blueberries and blueberry-based interventions for arterial stiffness.

Intervention	Location	Population of interest	Treatment Used	Subjects enrolled (subjects completed)	Treatment duration	Effects on arterial stiffness	Reference
**Freeze-dried blueberry powder**	King's College London, United Kingdom	Healthy men and women 65-80 years old	Treatment: 26 g of freeze-dried wild blueberry powder (302 mg anthocyanins)<br>Control: 26 g of matched placebo powder (0 mg anthocyanins)	Treatment: 35 (27)<br>Control: 31 (27)	12 weeks	-No changes or interaction for cfPWV<br>-No changes or interaction for AIx@HR75	Wood et al., 2023 [49]
**Blueberry mousse**	University of Milan, Italy	Healthy men and women ≥ 60 years old	Treatment: 250 g blueberry mousse (480 mg anthocyanins)<br>Control: 250 ml of sugar-matched water (0 mg anthocyanins)	Treatment: 20 (16)<br>Control: 20 (16)	Single acute dose	-No changes or interaction for AIx or AIx@HR75	Tucci et al., 2024 [50]
**Freeze-dried blueberry powder**	Florida State University, USA	Postmenopausal women 45-65 years old with pre- or stage 1 hypertension	Treatment: 22 g freeze-dried blueberry powder (470 mg anthocyanins)<br>Control: 22 g macronutrient-matched control powder (0 mg anthocyanins)	Treatment: 25 (20)<br>Control: 23 (20)	8 weeks	-No changes or interaction for cfPWV<br>-Significant reduction in baPWV in the treatment group with a group-by-time interaction	Johnson et al., 2015 [43]
**Fresh blueberry and freeze-dried blueberry powder**	Northumbria University, United Kingdom	Generally healthy normal and overweight men and women 18-60 years old	Treatment I: 160 g of fresh whole blueberry (221 mg polyphenols, anthocyanins NR)<br>Treatment II: 20 g of freeze-dried blueberry powder (289 mg polyphenols, anthocyanins NR)<br>Control: Capsule with plant-derived fiber microcrystalline cellulose (0 mg polyphenols, anthocyanins NR)	Treatment I: 42 (37)<br>Treatment II: 42 (37)<br>Control: 42 (37)	1 week	-No changes or interaction for crPWV	Wang et al., 2022 [45]
**Frozen thawed blueberry**	University of Milan, Italy	Healthy smoking and non-smoking men 20-30 years old with a Reactive Hyperemia Index < 1.67	Treatment I: 300 g of thawed frozen blueberry (309 mg anthocyanins)<br>Control: Sugar-matched control in 300 ml of water (0 mg anthocyanins)	Treatment: 24 (24)<br>Control: 24 (24)	Single acute dose	-No changes or interaction for AIx or AIx@HR75	Del Bo', et al., 2017 [51]
**Freeze-dried blueberry powder**	Colorado State University, USA	Postmenopausal women 45-65 years old with pre- or stage 1 hypertension	Treatment: 22 g freeze-dried highbush blueberry powder (224 mg anthocyanins)<br>Control: 22 g isocaloric and carbohydrate-matched placebo powder (224 mg anthocyanins)	Treatment: 24 (22)<br>Control: 24 (21)	12 weeks	-No changes or interaction for cfPWV<br>-No changes or interaction for AIx or AIx@HR75<br>-cPP was significantly reduced in the treatment group at 4 weeks compared to baseline, but not at 8 or 12 weeks; no other	Woolf et al., 2023 [48]

						significant differences in cBP	
**Freeze-dried blueberry powder**	Appalachian State University, USA	Postmenopausal women and men 18-50 years old	Treatment: 38 g freeze-dried blueberry powder (anthocyanins NR)<br>Control: powder containing maltodextrin, fructose, BB flavoring, coloring, citric acid, and silica (anthocyanins NR)	Treatment: 13 (13)<br>Control: 12 (12)	6 weeks	-No changes or interaction for cfPWV<br>-Reduction in AIx in the treatment group compared to baseline<br>-Reduction in cSBP in the treatment group compared to baseline; no other effects on cBP	McAnulty et al., 2014 [47]
**Freeze-dried blueberry powder**	University of East Anglia and Cambridge University, United Kingdom	Overweight and obese adults 50-75 years old with MetS	Treatment I: 26 g freeze-dried blueberries (384 mg anthocyanins)<br>Treatment II: 13 g freeze-dried blueberries, 13 g placebo material (182 mg anthocyanins)<br>Control: 26 g purple powder containing blueberry aromatics, dextrose, maltodextrin, fructose, artificial color and flavorings (0 mg anthocyanins)	Treatment I: 44 (37)<br>Treatment II: 49 (39)<br>Control: 45 (39)	6 months	-No changes or interaction for cfPWV<br>-AIx@HR75 significantly reduced in treatment I compared to treatment II and control groups	Curtis et al., 2019 [46]
**Frozen thawed blueberry**	University of Milan, Italy	Smoking men 20-30 years old	Treatment I: 300 g fresh-frozen blueberries (349 mg anthocyanins) + smoking<br>Treatment II: Smoking one cigarette (0 mg anthocyanins)<br>Control: 300 ml water with 16g fructose and 11g glucose (0 mg anthocyanins) + smoking	Treatment I: 16 (16)<br>Treatment II: 16 (16)<br>Control: 16 (16)	Single acute dose	-No changes found in AIx or AIx@HR75 with blueberry treatment, but a reduction in AIx occurred after Treatment II (smoking)	Del Bo' et al., 2014 [52]
**Freeze-dried blueberry powder**	Appalachian State University, USA	Men and women 40-70 years old	Treatment: 38 g freeze-dried blueberry powder (anthocyanins NR)<br>Control: placebo powder containing maltodextrin, fructose, blueberry flavoring, coloring, citric acid, silica (anthocyanins NR)	Treatment: 10 (10)<br>Control: 12 (12)	3 weeks	-No changes or interaction for cfPWV<br>-Significant time effects for AIx resulted, but no treatment or time by treatment interaction occurred	McAnulty et al., 2019 [53]
**Freeze-dried blueberry powder***	University of Milan, Italy	Men and women (details NR)	Treatment: 30 g of freeze-dried whole blueberry powder (459 mg of anthocyanins) in 300 ml of water<br>Control: Placebo drink (details NR)	Treatment: 20 (NR)<br>Control: 20 (NR)	Single acute dose	-Significant time effects resulted for AIx in a preliminary analysis of the first 12 subjects	Rendine et al., 2024 [54]
**Blueberry drink from freeze-dried blueberry powder**	University of Reading, United Kingdom	Healthy men 18-40 years old	Treatment I: Blueberry drink composed of 34 g freeze-dried blueberry powder (310 mg anthocyanins) + 500 ml low-nitrate water	Treatment I: 11 (10)<br>Treatment II: 11 (10)<br>Treatment III: 11 (10)<br>Control: 11 (10)	Single acute dose	-No changes or interaction for cfPWV<br>-No changes or interaction for AIx	Rodriguez-Mateos et al., 2013 [55]

			Treatment II: Blueberry drink composed of 57 g freeze-dried blueberry powder (517 mg anthocyanins) + 500 ml low-nitrate water<br>Treatment III: blueberry drink composed of 80 g freeze-dried blueberry powder (724 mg anthocyanins) + 500 ml low-nitrate water<br>Control: Macro- and micro-nutrient -matched control drink (0 mg anthocyanins)			-No changes or interaction for DVP-SI or DVP-RI<br>-No changes or interaction for cBP	
**Blueberry juice**	Northeast Normal University, China	Men and women 18-25 years old	Treatment I: low-intensity aerobic exercise<br>Treatment II: low-volume (25 ml) blueberry juice consumption group (168 mg of proanthocyanidins)<br>Treatment III: mid-volume (50 ml) blueberry juice consumption group (336 mg of proanthocyanidins)<br>Treatment IV: high-volume (100 ml) blueberry juice consumption group (672 mg of proanthocyanidins)<br>Treatment V: low-intensity aerobic exercise + low-volume (25 ml) blueberry juice consumption group (168 mg of proanthocyanidins)<br>Treatment VI: low-intensity aerobic exercise + mid-volume (50 ml) blueberry juice consumption group (336 mg of proanthocyanidins)<br>Treatment VII: low-intensity aerobic exercise + high-volume (100 ml) blueberry juice consumption group (672 mg of proanthocyanidins)<br>Control: No exercise or nutrition intervention	Treatment I: 6 (6)<br>Treatment II: 6 (6)<br>Treatment III: 6 (6)<br>Treatment IV: 6 (6)<br>Treatment V: 6 (6)<br>Treatment VI: 6 (6)<br>Treatment VII: 6 (6)<br>Control: 6 (6)	Single acute dose	-Significant main effect for time in baPWV, but no effect of groups or interaction	Wu et al., 2025 [44]

*Conference abstract only; AIx = augmentation index; AIx@HR75 = augmentation index standardized to a heart rate of 75 beats per minute; baPWV = brachial-ankle pulse wave velocity; cBP = central blood pressure; cfPWV = carotid-femoral pulse wave velocity; crPWV = carotid-radial pulse wave velocity; cPP = central pulse pressure; cSBP = central systolic blood pressure; DVP-RI = digital volume pulse reflection index; DVP-SI = digital volume pulse stiffness index; MetS = metabolic syndrome; NR = not reported

Table S2: Clinical trials considering aronia berries, blackberries, blackcurrants, cranberries, raspberries, strawberries, mixed berries, and berry-based interventions for arterial stiffness.

Intervention	Location	Population of interest	Treatment Used	Subjects enrolled (subjects completed)	Treatment duration	Effects on arterial stiffness	Reference
**Aronox® aronia berry extract**	King's College London, United Kingdom	Healthy prehypertensive men and women 40-70 years old	Treatment: 500 mg Aronox® aronia berry extract capsules (106 mg polyphenols)<br>Control: 500 mg carbohydrate-, calorie-, and fiber-matched maltodextrin capsules (0 mg polyphenols)	Treatment: 51 (45)<br>Control: 51 (48)	12 weeks	-Awake PWV was improved in treatment group after 12 weeks in a group-by-time interaction, but this change did not persist ~24 hr after the last capsule was taken<br>-24hr brachial and aortic AIx, as well as awake brachial and aortic AIx, were improved in the treatment group in a group-by-time interaction, but this improvement did not persist ~24 hr after the last capsule was taken<br>-No changes or interaction for cBP	Le Sayec et al., 2022 [59]
**Concentrated aronia berry extract and aronia berry whole fruit capsules**	King's College London, United Kingdom	Healthy men 18-45 years old	Treatment I: 500 mg concentrated aronia berry extract capsules made from 75g aronia berry minus fiber and organic acids (116 mg total polyphenols)<br>Treatment II: 500 mg aronia berry capsules made from 10g aronia whole fruit (12 mg total polyphenols)<br>Control: 500 mg maltodextrin (0 mg total polyphenols)	Treatment I: 23 (23)<br>Treatment II: 23 (23)<br>Control: 20 (20)	12 weeks + single acute dose	-No changes or interaction for cfPWV<br>-No changes or interaction for AIx<br>-No changes or interaction for cBP	Istas et al., 2019 [64]
**Unripe black raspberry extract**	Korea University Anam Hospital, South Korea	Men and women 18-75 years old with MetS	Treatment: 750 mg of unripe black raspberry powder (138 mg anthocyanins, 166 mg total polyphenols)<br>Control: Capsules containing isomaltose, magnesium stearate, and silica (polyphenols and anthocyanins NR)	Treatment: 26 (25)<br>Control: 25 (25)	12 weeks	-The change in AIx for the treatment was significantly different than the control group<br>-No changes or interaction for cBP	Jeong et al., 2016 [61]
**Blackberry polyphenol-enriched beverage***	University College Cork, Republic of Ireland	Men and women 45-75 years old with untreated elevated systolic BP	Treatment: 250 ml blackberry polyphenol-enriched beverage (700 mg GAE)<br>Control: 250 ml low-dose polyphenol beverage (<100 mg GAE)	Treatment: 83 (80)<br>Control: 83 (80)	6 weeks	-No changes or interaction for cBP	Heneghan et al., 2017 [65]
**CurraNZ® blackcurrant extract**	Nappon Sport Science University, Japan	Men and women ≥ 65 years old	Treatment: 600 mg CurraNZ® New Zealand blackcurrant extract capsules (210 mg anthocyanins)<br>Control: 600 mg microcrystalline cellulose (anthocyanins NR)	Treatment: 14 (14)<br>Control: 14 (14)	7 days	-cfPWV decreased significantly in the treatment group relative to the placebo group in a group-by-time interaction<br>-No changes or interaction for faPWV<br>-AIx decreased significantly in the treatment group, but no interaction occurred<br>-cSBP decreased significantly in the treatment group relative to the placebo	Okamoto et al., 2020 [58]

						group in a group-by-time interaction; no other changes in cBP	
**Freeze-dried cranberry powder**	University of Dusseldorf, Germany	Healthy men 18-45 years old	Treatment: 9 g freeze-dried cranberry powder (525 mg total polyphenols, 375 mg proanthocyanidins, 54 mg anthocyanins)<br>Control: 9 g placebo powder made from maltodextrin, citric acid, artificial cranberry flavor, fructose, red color, and grape shade (0 mg total polyphenols, 0 mg proanthocyanidins, 0 mg anthocyanins)	Treatment: 23 (22)<br>Control: 22 (22)	1 month	-No changes or interaction for cfPWV<br>-No changes or interaction for AIx	Heiss et al., 2022 [66]
**Double-strength cranberry juice**	Boston Medical Center, USA	Men and women with stable coronary artery disease	Treatment: 480 ml double-strength cranberry juice (835 mg total polyphenols, 94 mg anthocyanins)<br>Control: 480 ml calorie-matched placebo beverage containing no polyphenols (0 mg polyphenols, 0 mg anthocyanins)	Treatment: 47 (44)<br>Control: 47 (44)	4 weeks	-cfPWV was decreased by 0.5 m/s in the treatment group with a group-by-time interaction<br>-No changes or interaction for crPWV	Dohadwala et al., 2011 [57]
**Cranberry juice with differing amounts of total polyphenols**	University of Dusseldorf, Germany	Healthy men 18-35 years old	Treatment I: 450 ml cranberry juice (409 mg total polyphenols, 125 mg proanthocyanidins, 7 mg anthocyanins)<br>Treatment II: 450 ml cranberry juice (787 mg total polyphenols, 243 mg proanthocyanidins, 17 mg anthocyanins)<br>Treatment III: 450 ml cranberry juice (1238 mg total polyphenols, 279 mg proanthocyanidins, 24 mg anthocyanins)<br>Treatment IV: 450 ml cranberry juice (1534 mg total polyphenols, 421 mg proanthocyanidins, 27 mg anthocyanins)<br>Treatment V: 450 ml cranberry juice (1910 mg total polyphenols, 486 mg proanthocyanidins, 33 mg anthocyanins)<br>Control: 450 ml macro- and micro-nutrient isocaloric control drink (3 mg total polyphenols, 0 mg proanthocyanidins, 0 mg anthocyanins)	Treatment I: 10 (10)<br>Treatment II: 10 (10)<br>Treatment III: 10 (10)<br>Treatment IV: 10 (10)<br>Treatment V: 10 (10)<br>Control: 10 (10)	Single acute dose	-No changes or interaction for cfPWV<br>-No interaction for AIx, but a significant decrease in AIx from baseline was observed at 1.5 and 6 hr for Treatment IV and at 4 and 6 hr for Treatment I<br>-No interaction for cBP, but a significant decrease in cSBP from baseline was observed at 6 hr for Treatment V compared to baseline	Rodriguez-Mateos et al., 2016 [62]
**Cranberry juice**	Pennsylvania State University, USA	Men and women 30-65 years old with elevated blood pressure	Treatment: 500 ml cranberry juice (160 mg total polyphenols, 40 mg proanthocyanidins, 3 mg anthocyanins)<br>Control: 500 ml calorie- and carbohydrate-matched placebo juice (0 mg polyphenols, 0 mg proanthocyanidins, 0 mg anthocyanins)	Treatment: 47 (40)<br>Control: 47 (40)	8 weeks	-No changes or interaction for cfPWV<br>-No changes or interaction for AIx<br>-No changes or interaction for cBP	Richter et al., 2021 [67]
**Frozen red raspberry beverage**	University of Dusseldorf, Germany	Healthy men 18-35 years old	Treatment I: 592 ml beverage containing 400 g frozen red raspberries + 100 ml of water (403 mg total polyphenols, 328 mg anthocyanins)<br>Treatment II: 592 ml beverage containing 200 g frozen red raspberries + 300 ml of water + matched fiber, vitamin C, glucose, fructose, and citric acid (201 mg total polyphenols, 164 mg anthocyanins)	Treatment I: 10 (10)<br>Treatment II: 10 (10)<br>Control: 10 (10)	Single acute dose	-No changes or interaction for cfPWV<br>-No changes or interaction for AIx<br>-No changes or interaction for cBP	Istas et al., 2018 [68]

			Control: 592 ml placebo drink micro- and macronutrient-matched to the 400 g raspberry drink, made by blending soluble and insoluble fibers, sugars, citric acid, red colorant, synthetic raspberry flavor, water (0 mg total polyphenols, 0 mg anthocyanins)				
**Mixed red berry fruit juice**	German Sport University Cologne, Germany	Untrained pre-diabetic men and women 40-70 years old	Treatment: 200 ml red berry fruit juice (820 mg total polyphenols)<br>Control: 200 ml isocaloric placebo drink with similar potassium and sugar but lower polyphenols (19 mg total polyphenols)	Treatment: 13 (10)<br>Control: 13 (10)	14 days	-Significant time effects resulted for PWV, but no treatment group-by-time interaction occurred	Valder et al., 2025 [63]
**Freeze-dried strawberry powder**	Pennsylvania State University, USA	Overweight and obese men and women 35-60 years old	Treatment I: 40 g "high dose" freeze-dried strawberry powder (40 g freeze-dried strawberry powder) (total polyphenols, anthocyanins NR)<br>Treatment II: 40 g "low dose" freeze-dried strawberry powder (13 g freeze-dried strawberry powder, 27 g placebo powder) (total polyphenols, anthocyanins NR)<br>Control: 40 g fiber-matched placebo powder (0 g freeze-dried strawberry powder, 40 mg placebo powder) (total polyphenols, anthocyanins NR)	Treatment: 42 (40)<br>Control: 42 (40)	4 weeks	-No changes or interaction for PWV<br>-No changes or interaction for AIx<br>-No changes or interaction for cBP	Richter et al., 2023 [69]
**Freeze-dried strawberry powder**	Florida State University, USA	Postmenopausal women 45-65 years old with prehypertension or stage 1 hypertension	Treatment I: 50 g freeze-dried strawberry powder (248 mg total polyphenols, 205 mg anthocyanins)<br>Treatment II: 25 g freeze-dried strawberry powder (124 mg total polyphenols, 103 mg anthocyanins)<br>Control: 25 g placebo powder made from fructose, sucrose, dextrose, tapioca maltodextrin, modified food starch, erythritol, cellulose gum, citric acid, xanthan gum, malic acid, sugar beet fiber, silicon dioxide, natural strawberry flavor, and color additives (0 mg total polyphenols, 0 mg anthocyanins)	Treatment I: 24 (20)<br>Treatment II: 25 (20)<br>Control: 22 (20)	8 weeks	-baPWV and faPWV were reduced from baseline in treatment II group, but no group-by-time interaction occurred	Feresin et al., 2017 [60]

*Conference abstract only; AIx = augmentation index; baPWV = brachial-ankle pulse wave velocity; cBP = central blood pressure; cfPWV = carotid-femoral pulse wave velocity; crPWV = carotid-radial pulse wave velocity; cSBP = central systolic blood pressure; faPWV = femoral-ankle pulse wave velocity; GAE = gallic acid equivalents; MetS = metabolic syndrome; NR = not reported; PWV = pulse wave velocity

Table S3: Clinical trials considering tart cherries and tart cherry-based interventions for arterial stiffness.

Intervention	Location	Population of interest	Treatment Used	Subjects enrolled (subjects completed)	Treatment duration	Effects on arterial stiffness	Reference
**CherryActive® Montmorency tart cherry concentrate**	Northumbria University, United Kingdom	Men with early hypertension	Treatment: 60 ml CherryActive® Montmorency tart cherry concentrate (74 mg anthocyanins) + 100 ml water<br>Control: 60 ml fruit-flavored cordial (0 mg anthocyanins) + 100 ml water	Treatment: 16 (15)<br>Control: 16 (15)	Single acute dose	-A time effect was present in cfPWV, but no interaction for cfPWV<br>-No changes or interaction for AIx or AIx@HR75<br>-A time effect was present in DVP-RI, but no interaction for DVP-RI<br>-No changes or interaction for DVP-SI	Keane et al., 2016 [70]
**Montmorency tart cherry juice**	Colorado State University, USA	Men and women 20-60 years old with MetS	Treatment: 480 ml tart cherry juice (88 mg anthocyanins)<br>Control: 480 ml calorie-matched placebo-control drink (0 mg anthocyanins)	Treatment: 13 (9)<br>Control: 13 (10)	12 weeks	-No changes or interaction for cfPWV, baPWV, or faPWV<br>-No changes or interaction for AIx or AIx@HR75<br>-No changes or interaction for cBP or AP	Johnson et al., 2020 [74]
**Montmorency tart cherry**	Northumbria University, United Kingdom	Men and women 40-60 years old with risk factors for type 2 diabetes	Treatment: 60 ml of Montmorency tart cherry in 440 ml of water (68-74 mg anthocyanins)<br>Control: 60 ml of unsweetened black cherry flavored Kool-aid in 480 ml of water (0 mg anthocyanins)	Treatment: 28 (25)<br>Control: 28 (25)	3 months	-No changes or interaction for cfPWV<br>-No changes or interaction for AIx or AIx@HR75	Kimble et al., 2021 [75]
**CherryActive® Montmorency tart cherry concentrate**	Northumbria University, United Kingdom	Healthy men and women	Treatment: 60 ml CherryActive® Montmorency tart cherry concentrate (74 mg of anthocyanins) + 200 ml water<br>Control: 260 ml low fruit (<1 %) unsweetened black cherry cordial mixed with ingredients to match MC concentrate for energy and macronutrients (anthocyanins NR)	Treatment: NR (12)<br>Control: NR (11)	4 weeks	-No changes or interaction for PWV<br>-No changes or interaction for DVP-SI or DVP-RI	Kimble et al., 2021 [76]
**CherryActive® Montmorency tart cherry concentrate or capsules**	University of Hertfordshire, United Kingdom	Men and women with MetS	Treatment I: 30 ml Montmorency tart cherry concentrate (270 mg anthocyanins) + 100 ml water<br>Treatment II: 10 capsules of freeze-dried Montmorency tart cherry skin powder (270 mg anthocyanins) + 130 ml water<br>Control: 30 ml energy-matched commercially available fruit-flavored cordial (0 mg anthocyanins) + 100 ml water + maltodextrin	Treatment: 13 (11)<br>Control: 13 (11)	Single acute dose	-A main effect for time was detected for AIx and AIx@HR75, but no treatment or interaction; post-hoc comparisons revealed a significant difference between placebo and Treatment I in AIx at 2 hr but not 30 min, 1 hr, 3 hr, 4 hr, or 5 hr<br>-No changes or interaction for cBP or cPP<br>-A main effect for time was detected for AP, but no treatment or interaction; post-hoc comparisons revealed a significant difference between placebo and Treatment I in AP at 2 hr but not 30 min, 1 hr, 3 hr, 4 hr, or 5 hr	Desai et al., 2019 [72]
**CherryActive® Montmorency tart cherry concentrate**	University of Hertfordshire, United Kingdom	Men and women with MetS	Treatment I: 30 ml Montmorency tart cherry concentrate (270 mg anthocyanins) + 100 ml water	Treatment: 15 (12)<br>Control: 15 (12)	7 days	-No changes or interaction for AIx<br>-No changes or interaction for AIx@HR75 after the 7-day treatment,	Desai et al., 2021 [71]

			Control: 30 ml energy-matched commercially available fruit-flavored cordial + 100 ml water + dextrose + fructose + corn flour + citric acid (0 mg anthocyanins)			but an acute main effect of condition was found on AIx@HR75<br>-No changes or interaction for cBP or bPP<br>-No changes or interaction for AP	
**CherryActive® Montmorency tart cherry concentrate**	Sheffield Hallam University, United Kingdom	Healthy men and women 30-50 years old	Treatment: 30 ml Cherry Active® cherry juice concentrate (274 mg anthocyanins) diluted with 220 ml of water<br>Control: 250 ml Sprite® control drink composed of lemonade (0 mg anthocyanins)	Treatment: 26 (25)<br>Control: 21 (21)	6 weeks	-No changes or interaction for bkPWV	Lynn et al., 2014 [31]
**CherryActive® Montmorency tart cherry concentrate**	Sheffield Hallam University, United Kingdom	Healthy men and women 18-85 years old	Treatment: 30 ml Cherry Active® cherry juice concentrate (3.8 mg cyanidin-3-glucoside equivalents) diluted with 220 ml of low-nitrate water (Buxton®)<br>Control: 250 ml of low-nitrate water (Buxton®) (0 mg cyanidin-3-glucoside equivalents)	Treatment: 13 (12)<br>Control: 13 (12)	Single acute dose	-No changes or interaction for cfPWV<br>-No changes or interaction for AIx<br>-A main effect for time was detected for cSBP and cDBP, but no treatment or interaction	Lamb et al., 2026 [73]

AIx = augmentation index; AIx@HR75 = augmentation index standardized to a heart rate of 75 beats per minute; AP = augmentation pressure; baPWV = brachial-ankle pulse wave velocity; bkPWV = brachial-knee pulse wave velocity; cBP = central blood pressure; cDBP = central diastolic blood pressure; cfPWV = carotid-femoral pulse wave velocity; cPP = central pulse pressure; cSBP = central systolic blood pressure; DVP-RI = digital volume pulse reflection index; DVP-SI = digital volume pulse stiffness index; faPWV = femoral-ankle pulse wave velocity; NR = not reported; PWV = pulse wave velocity

Table S4: Clinical trials considering grape-based interventions for arterial stiffness.

Intervention	Location	Population of interest	Treatment Used	Subjects enrolled (subjects completed)	Treatment duration	Effects on arterial stiffness	Reference
**Welch's Concord grape juice**	University of Athens Medical School, Greece	Men and women smokers	Treatment: 7 cc/kg of Welch's Concord grape juice (473 mg total polyphenols per 240 ml)<br>Control: 7 cc/kg of grapefruit juice (0 mg total polyphenols per 240 ml)	Treatment: 26 (NR)<br>Control: 26 (NR)	14 days	-Reduction in cfPWV in the treatment group compared to baseline, but no interaction; treatment also prevented the acute smoking-induced increase in cfPWV at days 7 and 14	Siasos et al., 2014 [77]
**Welch's Concord grape juice**	Boston University School of Medicine, USA	Men and women with upper range prehypertension or stage 1 hypertension	Treatment: 7 ml/kg Concord grape juice (473 mg total polyphenols per 240 ml)<br>Control: 7 ml/kg sugar- and calorie-matched Concord grape-flavored placebo beverage (0 mg total polyphenols)	Treatment: 83 (64)<br>Control: 83 (64)	8 weeks	-No changes or interaction for cfPWV or crPWV<br>-No changes or interaction for bPP	Dohadwala et al., 2010 [79]
**Meganatural®-BP grape seed extract**	California Baptist University, USA	Obese and normal weight men 18-29 years old	Treatment: 300 mg Meganatural® grape seed extract (286 total polyphenols)<br>Control: 300 mg starch (total polyphenols NR)	Treatment: 10 (NR)<br>Control: 10 (NR)	Single acute dose	-No changes or interaction for aortic stiffness measured by bioelectrical impedance	Dillon et al., 2020 [80]
**Gravinol™ grape seed proanthocyanidin extract**	Tokyo Medical and Dental University, Japan	Prehypertensive men and women 40-64 years old	Treatment I: 200 mg Gravinol™ "low dose" grape seed extract (~170 mg proanthocyanidins)<br>Treatment II: 400 mg Gravinol™ "high dose" grape seed extract (~340 mg proanthocyanidins)	Treatment I: 10 (10)<br>Treatment II: 10 (10)<br>Control: 10 (10)	12 weeks	-PWV improved with Treatment II at the 8- and 12-week marks compared to baseline, but there was no group-by-time interaction	Odai et al., 2019 [78]

			Control: 0 mg grape seed extract placebo (contents NR)				
**Grape seed polyphenols**	University of Western Australia, Australia	Hypertensive men and women on antihypertensive drugs for 3 or more months	Treatment I: 500 mg/d of vitamin C and matched grape seed polyphenols placebo (total polyphenols NR)<br>Treatment II: 1000 mg/d of grape seed polyphenols (1000 mg total polyphenols) and matched vitamin C placebo<br>Treatment III: 500 mg/d of vitamin C and 1000 mg/d of grape seed polyphenols (1000 mg total polyphenols)<br>Control: matched placebo tablets for both grape seed polyphenols and vitamin C (total polyphenols NR)	Treatment I: 19 (19)<br>Treatment II: 18 (16)<br>Treatment III: 18 (16)<br>Control: 19 (18)	6 weeks	-No significant differences occurred with Treatment II alone, but Treatment III resulted in a higher rate of nighttime bPP variation (detrimental effect)	Hodgson et al., 2014 [81]
**Raisins**	Harokopio University, Greece	Men and women smokers 20-40 years old	Treatment: Normal smoking habits + 90 g raisins (total polyphenols NR)<br>Control: Normal smoking habits	Treatment: 22 (20)<br>Control: 14 (13)	4 weeks	-No changes or interaction for cfPWV or crPWV	Kanellos et al., 2017 [82]

bPP = brachial pulse pressure; cfPWV = carotid-femoral pulse wave velocity; crPWV = carotid-radial pulse wave velocity; GAE = gallic acid equivalents; NR = not reported; PWV = pulse wave velocity

Table S5: Clinical trials considering citrus-based interventions for arterial stiffness.

Intervention	Location	Population of interest	Treatment Used	Subjects enrolled (subjects completed)	Treatment duration	Effects on arterial stiffness	Reference
**Blood orange juice**	Quadram Institute Bioscience, United Kingdom	Men and women 25-84 years old	Treatment: 500 ml blood orange juice (50 mg anthocyanins)<br>Control: 500 ml standard orange juice (0 mg anthocyanins)	Treatment: 45 (41)<br>Control: 45 (41)	28 days	-No changes or interaction for cfPWV or baPWV<br>-No changes or interaction for cBP	Hollands et al., 2018 [87]
**Grapefruit juice**	University Hospital of Clermont-Ferrand, France	Healthy postmenopausal Caucasian women 50-65 years old	Treatment: 340 ml grapefruit juice (213 mg naringenin glycosides)<br>Control: 340 ml macro- and micronutrient matched control drink (0 mg naringenin glycosides)	Treatment: 52 (48)<br>Control: 52 (48)	6 months	-cfPWV was lower in the treatment group compared to placebo when adjusted for baseline values<br>-No changes or interaction for AIX@HR75	Habauzit et al., 2015 [83]
**Concentrated β-cryptoxanthin-rich Satsuma mandarin juice**	Hamamatsu University School of Medicine, Japan	Men and women 25-74 years old	Treatment: 125 ml concentrated β-cryptoxanthin-rich (4 mg) Satsuma mandarin juice (hesperidin NR)<br>Control: 125 ml β-cryptoxanthin-deprived (0 mg) Satsuma mandarin juice (hesperidin NR)	Treatment: 59 (50)<br>Control: 59 (50)	12 weeks	-No significant interaction for baPWV during the time the intervention transpired; however, baPWV had increased in both the treatment and control groups 8 months post-intervention, but a significant group-by-time interaction in baPWV occurred, revealing a significantly lower baPWV in the treatment group	Nakamura et al., 2017 [84]
**Orange juice and hesperidin-enriched orange juice**	Hospital Universitari Sant Joan, Spain	Men and women 18-65 years old with pre- or	Treatment I: 500 ml orange juice (345 mg hesperidin)<br>Treatment II: 500 ml hesperidin-enriched orange juice (600 mg hesperidin)	Treatment I: 53 (46)<br>Treatment II: 53 (40)	12 weeks	-bPP decreased in a dose-dependent manner relative to the hesperidin content of the treatment beverages at 2 weeks, 6 weeks, and 10 weeks, but the linear trend	Valls et al., 2020 [85]

		stage 1 hypertension	Control: 500 ml control drink (contents NR, 0 mg hesperidin)	Control: 53 (43)		did not reach significance at 12 weeks; bPP was significantly reduced at 2 hr, 4 hr, and 6 hr after a single acute dose of treatment II only	
**Orange juice and hesperidin supplement**	University of East Anglia, United Kingdom	Healthy men 51-69 years old	Treatment I: Hesperidin supplement (320 mg hesperidin)<br>Treatment II: 767 ml orange juice (320 mg hesperidin)<br>Control: 767 ml sugar-, Vitamin C-matched placebo drink	Treatment: 16 (14)<br>Control: 16 (14)	Single acute dose	-No changes or interaction for cfPWV<br>-No changes or interaction for AIx@HR75	Schar et al., 2015 [86]

AIx@HR75 = augmentation index standardized to a heart rate of 75 beats per minute; baPWV = brachial-ankle pulse wave velocity; bPP = brachial pulse pressure; cBP = central blood pressure; cfPWV = carotid-femoral pulse wave velocity; NR = not reported

Table S6: Clinical trials considering watermelon-based interventions for arterial stiffness.

Intervention	Location	Population of interest	Treatment Used	Subjects enrolled (subjects completed)	Treatment duration	Effects on arterial stiffness	Reference
**Freeze-dried watermelon extract**	Florida State University, USA	Postmenopausal women	Treatment: 6 g freeze-dried watermelon extract (2:1 L-citrulline/L-arginine)<br>Control: Sugar-matched placebo powder (sucrose, glucose, and fructose at 2:2:1)	Treatment: 12 (12)<br>Control: 12 (12)	6 weeks	-baPWV decreased significantly in the treatment group in a group-by-time interaction<br>-No changes or interaction for AIx or AIx@HR75<br>-cSBP and cDBP decreased significantly with treatment in a group-by-time interaction	Figueroa et al., 2013 [88]
**Wild watermelon juice**	Ritsumeikan University, Japan	Young, healthy women	Treatment: 100 ml wild watermelon-extracted juice with 90 g of watermelon extract (162 mg L-citrulline, 30 mg L-arginine)<br>Control: 100 ml placebo beverage with citric acid and lemon fragrance oil (0 mg citrulline, 0 mg arginine)	Treatment: 12 (NR)<br>Control: 12 (NR)	Single acute dose	-No changes or interaction for cfPWV or baPWV<br>-A significant interaction in the percentage change in faPWV, with a significant reduction at 30 min, but no further changes at 60 and 90 min	Fujie et al., 2022 [89]
**Freeze-dried watermelon extract**	Florida State University, USA	Prehypertensive, middle-aged men and women	Treatment: 4 g freeze-dried watermelon powder (L-citrulline/ L-arginine: 2.7 g/1.3 g)<br>Control: Sugar-matched placebo powder (sucrose, glucose, and fructose at 2:2:1)	Treatment: 9 (9)<br>Control: 9 (9)	6 weeks	-No changes or interaction for cfPWV<br>-The difference between the treatment and control groups in AIx and AIx@HR75 was significantly larger at 6 weeks than baseline<br>-The difference between the treatment and control groups in bPP, cPP and cSBP was significantly larger at 6 weeks than baseline	Figueroa et al., 2011 [91]
**Freeze-dried watermelon extract**	Florida State University, USA	Middle-aged, obese men and women with pre- or stage 1 hypertension and normal ankle-brachial index	Treatment: 6 g freeze-dried watermelon powder (L-citrulline/ L-arginine: 2:1)<br>Control: Sugar-matched placebo powder (sucrose, glucose, and fructose at 2:2:1)	Treatment: 14 (14)<br>Control: 14 (14)	5 weeks	-Significant reduction in AIx in a group-by-time interaction	Figueroa et al., 2012 [90]

**Watermelon juice**	University of Alabama, USA	Non-obese postmenopausal women 55-70 years old	Treatment: 720 ml of 100% watermelon juice (1.6 g L-citrulline/1.2 g L-arginine)<br>Control: Isocaloric placebo with water, sucrose, watermelon flavoring, malic acid, pectin cellulose, and food coloring	Treatment: 21 (17)<br>Control: 21 (17)	4 weeks	-No changes or interaction for PWV<br>-No changes or interaction for bPP	Ellis et al., 2021 [92]

AIx = augmentation index; AIx@HR75 = augmentation index standardized to a heart rate of 75 beats per minute; baPWV = brachial-ankle pulse wave velocity; bPP = brachial pulse pressure; cDBP = central diastolic blood pressure; cfPWV = carotid-femoral pulse wave velocity; cPP = central pulse pressure; cSBP = central systolic blood pressure; faPWV = femoral-ankle pulse wave velocity; NR = not reported; PWV = pulse wave velocity

Table S7: Clinical trials considering other fruit and fruit-based interventions for arterial stiffness.

Intervention	Location	Population of interest	Treatment Used	Subjects enrolled (subjects completed)	Treatment duration	Effects on arterial stiffness	Reference
**Apple flavanol extract**	Quadram Institute Bioscience, United Kingdom	Apparently healthy men and women ≥ 50 years old with SBP 120-159 mmHg	Treatment I: single dose apple flavanol extract (70 mg monomeric flavanols, 65 mg procyanidins)<br>Treatment II: double dose apple flavanol extract (140 mg monomeric flavanols, 130 mg procyanidins)<br>Treatment III: apple procyanidin extract (6.5 mg monomeric flavanols, 130 mg procyanidins)<br>Control: microcrystalline cellulose	Treatment I: 43 (42)<br>Treatment II: 43 (42)<br>Treatment III: 43 (42)<br>Control: 43 (42)	Single acute dose + 4 weeks	-No changes or interaction for cfPWV or baPWV acutely or after 4-week intervention<br>-No changes or interaction for AIx or cBP acutely or after 4-week intervention	Hollands et al., 2018 [100]
**Fresh skins of pink apples**	University of Western Australia, Australia	Men and women at risk for CVD	Treatment: one whole Cripps Pink apple with skin + skin only for a second Cripps Pink apple, blended with water (244 mg flavanols, 6 mg anthocyanins)<br>Control: flesh only of one Cripps pink apple, blended with water (38 mg flavanols, 0 mg anthocyanins)	Treatment: 36 (30)<br>Control: 36 (30)	Single acute dose + 4 weeks	-No changes or interaction for PWV acutely or after 4-week intervention<br>-No changes or interaction for AIx@HR75 or cDBP (cSBP NR) acutely or after 4-week intervention	Bondonno et al., 2017 [101]
**Fresh apples**	University of Reading, United Kingdom	Generally healthy men and women 29-69 years old	Treatment: 340 g serving of apples, or 2 apples (990 mg total polyphenols)<br>Control: 500 ml sugar-matched apple juice concentrate beverage (3 mg total polyphenols)	Treatment: 43 (40)<br>Control: 43 (40)	8 weeks	-No changes or interaction for AIx@HR75<br>-No changes or interaction for cBP	Koutsos et al., 2020 [102]
**Fresh avocado**	Pennsylvania State University, USA	Men and women ≥ 25 years old with abdominal obesity	Treatment: 1 avocado + habitual diet (polyphenols NR)<br>Control: habitual diet (limited to 2 or less avocados per month) (polyphenols NR)	Treatment: 67 (65)<br>Control: 67 (64)	6 months	-No changes or interaction for cfPWV<br>-No changes or interaction for AIx<br>-No changes or interaction for cBP	Davis et al., 2024 [103]
**Fresh avocado and mango**	Illinois Institute of Technology, USA	Overweight and obese, prediabetic, middle-aged men and women	Treatment: Partially controlled diet reflecting the typical American diet + 1 Hass avocado + 1 cup mango daily (polyphenols NR)<br>Control: Partially controlled diet reflecting the typical American diet + equicaloric	Treatment: 41 (30)<br>Control: 41 (38)	8 weeks	-No changes or interaction for cfPWV<br>-No changes or interaction for cBP	Preiss et al., 2026 [104]

			carbohydrate food alternatives for the avocado and mango (polyphenols NR)				
**Freeze-dried dragon fruit**	King's College London, United Kingdom	Healthy men and women 18-40 years old	Treatment: 24 g freeze-dried red dragon fruit pulp powder (34 mg betalains, 10 mg total polyphenols)<br>Control: 24 g nutrient-matched placebo powder (betalains and total polyphenols NR)	Treatment (acute): 20 (19)<br>Control (acute): 20 (19)<br>Treatment: 20 (18)<br>Control: 20 (18)	Single acute dose + 14 days	-Significant decrease in cfPWV of 0.5 m/s at 3 h post-consumption compared to placebo, but no changes or interaction in cfPWV at 14 days<br>-AIx@HR75 did not show acute changes, but there was a 7% decrease in the treatment group relative to the placebo group at 14 days<br>-No changes or interaction for cBP acutely or after 14 days	Cheok et al., 2022 [93]
**Acanthopanax senticosus Harms fruit extract**	Ewha Womans University, South Korea	Men and women 20-64 years old with elevated risk for CVD	Treatment I: 1,000 mg Acanthopanax senticosus Harms fruit ("high dose") capsule (polyphenols NR)<br>Treatment II: 500 mg Acanthopanax senticosus Harms fruit ("low dose") capsule (polyphenols NR)<br>Control: placebo capsule with 0 mg Acanthopanax senticosus Harms fruit (polyphenols NR)	Treatment I: 34 (25)<br>Treatment II: 33 (26)<br>Control: 31 (25)	12 weeks	-Significant group-by-time interaction showing a reduction in baPWV with Treatment II relative to the placebo group	Oh et al., 2020 [95]
**Mixed dried fruit**	Pennsylvania State University, USA	Overweight and obese men and women 25-60 years old with at least one other cardiometabolic risk factor	Treatment: 112g of dried fruit consisting of 28g dried plums, 28g, Black Mission figs, 28g Deglet Noor dates, and 28g raisins (41 mg total polyphenols)<br>Control: 43g animal crackers + 51g fruit snack gummies (energy- and carbohydrate-matched) (5 mg total polyphenols)	Treatment: 55 (52)<br>Control: 55 (52)	4 weeks	-No changes or interaction for cfPWV<br>-No changes or interaction for AIx<br>-No changes or interaction for AP<br>-cDBP increased for the control condition only, but no other changes were found in cBP	Sullivan et al., 2020 [105]
**Proliva® olive fruit extract**	Euromed, Spain	Men and women 45-65 years old	Treatment I: 250 mg Proliva® standard olive fruit extract (50 mg hydroxytyrosol)<br>Treatment II: 500 mg Proliva ® standard olive fruit extract (100 mg hydroxytyrosol)<br>Control: contents NR (hydroxytyrosol NR)	Treatment I: 12 (NR)<br>Treatment II: 12 (NR)<br>Control: 12 (NR)	11 days	-CAVI scores were reduced in treatment and control groups, but no interaction or differences between groups were present	Pais et al., 2016 [106]
**Fresh pear**	Florida State University, USA	Men and women 45-65 years old with MetS	Treatment: 2 medium sized fresh pears daily (polyphenols NR)<br>Control: 50 g of calorie-matched control drink powder (maltodextrin, artificial and natural flavoring, artificial color, citric acid, and silica dioxide) (polyphenols NR)	Treatment: 50 (40)<br>Control: 50 (40)	12 weeks	-bPP was significantly reduced in treatment group at 6 and 12 weeks compared to baseline, but no interaction occurred	Navaei et al., 2019 [98]
**Pomegranate juice**	Western Galilee Hospital, Israel	Men and women >18 years on chronic dialysis treatment	Treatment: 100 ml pomegranate juice (120 mg of total polyphenols)<br>Control: 100 ml matched placebo juice with artificial pomegranate extract, citric acid, caramel, and sugar substitutes (0 mg of total polyphenols)	Treatment: 66 (41)<br>Control: 35 (26)	One year	-bPP was significantly reduced in the treatment group from baseline by 8.8%, but there was no group-by-time interaction	Shema-Didi, et al., 2014 [99]
**Pomegranate Pomanox® P30 extract**	Queen Margaret University, United Kingdom	Men and women 18-65 years old	Treatment: 1.08 g capsule of Pomanox® P30 whole pomegranate fruit extract (328 mg total polyphenols, 1 mg anthocyanins)<br>Control: 1.08 g capsule of maltodextrin (total polyphenols and anthocyanins NR)	Treatment: 12 (12)<br>Control: 12 (12)	28 days	-cfPWV was significantly reduced in the treatment group from baseline, but no interaction was apparent (p value for interaction NR)	Al-Dujaili et al., 2022 [94]

**Pomegranate juice**	University of Sheffield, United Kingdom	Healthy men and women 30-50 years old	Treatment: 330 ml pomegranate juice (1045 mg total polyphenols)<br>Control: 330 ml lemonade (total polyphenols NR)	Treatment: 26 (24)<br>Control: 25 (24)	4 weeks	-No changes or interaction for PWV	Lynn et al., 2012 [107]
**Pomegranate juice**	Queen Margaret University, United Kingdom	Overweight and obese men and women 40-65 years old with sedentary to moderate activity levels	Treatment: 500 ml pomegranate juice (843 mg polyphenols)<br>Control: 500 ml carbohydrate-matched placebo drink (total polyphenols NR)	Treatment: NR (28)<br>Control: NR (28)	4 weeks	-PWV was significantly reduced in the treatment group at 2 weeks and 4 weeks compared to baseline, but no interaction was apparent (p value for interaction NR)	Tsang et al., 2012 [96]
**Dried plums (prunes)**	University of California, USA	Healthy postmenopausal women 50-70 years old	Treatment: 6 dried plums (prunes), or the standard serving size (total polyphenols NR)<br>Control: 2 dried prunes (total polyphenols NR)	Treatment: 27 (27)<br>Control: 27 (27)	2 weeks	-No changes or interaction for AIx or AIx@HR75	Al-Dashti et al., 2019 [108]
**Fruitflow® tomato powder**	University of Oslo, Norway	Apparently healthy men 25-65 years old	Treatment: 150 mg Fruitflow® powder (up to 9 mg nucleotide derivatives, up to 10 mg phenolic conjugates, up to 7 mg flavonoid derivatives)<br>Control: Microcrystalline cellulose (polyphenols NR)	Treatment: 18 (12)<br>Control: 18 (12)	Single acute dose	-bPP was lower in the treatment group over 24-h time course than the placebo<br>-AIx tested in a subgroup (n = 6) was lowered by 8% in the treatment group compared to placebo over 24-h time course	Uddin et al., 2018 [97]
**Tomato-based foods**	University of Aberdeen, United Kingdom	Men and women 40-65 years old with BMI 18.5-35	Treatment I: Diet low in tomato-based foods + 10 mg lycopene (polyphenols NR)<br>Treatment II: High tomato-based products (corresponding to ~10 mg lycopene) (polyphenols NR)<br>Control: Diet low in tomato-based foods (polyphenols NR)	Treatment I: 79 (68)<br>Treatment II: 84 (81)<br>Control: 81 (76)	16 weeks	-No changes or interaction for PWV	Thies et al., 2012 [109]
**Tomato paste***	Instituto Politécnico de Coimbra, Portugal	Healthy, young sedentary men and women	Treatment: 70g tomato paste (polyphenols NR)<br>Control: NR (polyphenols NR)	Treatment: 30 (NR)<br>Control: 30 (NR)	4 weeks	-No changes or interaction for cfPWV<br>-No changes or interaction for AIx<br>-No changes or interaction for cBP	Pereira et al., 2015 [110]

*Conference abstract only; AIx = augmentation index; AIx@HR75 = augmentation index standardized to a heart rate of 75 beats per minute; AP = augmentation pressure; baPWV = brachial-ankle pulse wave velocity; bPP = brachial pulse pressure; CAVI = cardio-ankle vascular index; cBP = central blood pressure; cDBP = central diastolic blood pressure; cfPWV = carotid-femoral pulse wave velocity; cSBP = central systolic blood pressure; CVD = cardiovascular disease; DVP-RI = digital volume pulse reflection index; DVP-SI = digital volume pulse stiffness index; GAE = gallic acid equivalents; NR = not reported; PWV = pulse wave velocity
